# Spatio-temporal simulations of bone remodelling using a bone cell population model based on cell availability

**DOI:** 10.3389/fbioe.2023.1060158

**Published:** 2023-03-07

**Authors:** José Luis Calvo-Gallego, Pablo Manchado-Morales, Peter Pivonka, Javier Martínez-Reina

**Affiliations:** ^1^ Departamento de Ingeniería Mecánica y Fabricación, Universidad de Sevilla, Seville, Spain; ^2^ School of Mechanical, Medical and Process Engineering, Queensland University of Technology, Brisbane, QLD, Australia

**Keywords:** basic multicellular units, resorption period, formation period, BMU activation frequency, targeted bone remodelling, TGF-β, reversal period, BMU coupling

## Abstract

Here we developed a spatio-temporal bone remodeling model to simulate the action of Basic Multicelluar Units (BMUs). This model is based on two major extensions of a temporal-only bone cell population model (BCPM). First, the differentiation into mature resorbing osteoclasts and mature forming osteoblasts from their respective precursor cells was modelled as an intermittent process based on precursor cells availability. Second, the interaction between neighbouring BMUs was considered based on a “metabolic cost” argument which warrants that no new BMU will be activated in the neighbourhood of an existing BMU. With the proposed model we have simulated the phases of the remodelling process obtaining average periods similar to those found in the literature: resorption ( ∼22 days)—reversal (∼8 days)—formation (∼65 days)—quiescence (560–600 days) and an average BMU activation frequency of ∼1.6 BMUs/year/mm^3^. We further show here that the resorption and formation phases of the BMU become coordinated only by the presence of TGF-β (transforming growth factor *β*), i.e., a major coupling factor stored in the bone matrix. TGF-β is released through resorption so upregulating osteoclast apoptosis and accumulation of osteoblast precursors, i.e., facilitating the transition from the resorption to the formation phase at a given remodelling site. Finally, we demonstrate that this model can explain targeted bone remodelling as the BMUs are steered towards damaged bone areas in order to commence bone matrix repair.

## 1 Introduction

Bone adapts itself to the surrounding mechanobiological environment to maintain its stiffness, strength, weight, material and cellular composition in an optimum state that allows it to perform its functions properly. This adaptation occurs in two ways: at the surfaces, either endosteum or periosteum, in the latter case modifying bone size and shape, in a process known as bone modelling; in the interior where it mainly modifies porosity and anisotropy, repairs microstructural damage and contributes to regulate calcium homeostasis. This is achieved by the spatio-temporal action of bone cells, commonly referred to as Basic Multicellular Unit (BMU) (Martin et al., 2015), that resorb and form bone in packets, thus leading to the “quantum concept of bone remodelling” (Parfitt, 1979). Understanding the appearance of BMUs and its cyclic activity is essential to extend our knowledge of normal bone physiology and its disorders; for instance, about the lengths of the different phases of the BMU, the coupling between these phases and the factors that control that coupling. The knowledge of these aspects can be fundamental to explain the abnormal behaviours observed in certain bone diseases.

The life of a BMU begins when osteoclasts precursors differentiate and fuse to form multinucleated mature osteoclasts at a given bone site. These mature cells first solubilize the mineral by secreting acids and then break down the exposed demineralized collagen by secreting lysosomal collagenolytic cysteine-proteinases within their ruffled borders (Vaes et al., 1992), thus creating a resorption cavity (Howship’s lacunae) which can grow in certain directions (Burger et al., 2003) due to the high motility of osteoclasts. Some time after resorption has ceased at a given location osteoblasts precursors differentiate into active forming osteoblasts. These cells secrete osteoid, mainly composed of collagen and water and later mineralised, and follow osteoclasts, thus refilling the resorption cavity and closing the bone remodelling (BR) cycle (Martin et al., 2015).

The lapse between these catabolic and anabolic phases, called reversal phase, can last from 8–9 days (Eriksen et al., 1984; Hazelwood et al., 2001) to several weeks (Delaisse, 2014; Martin, 2021). First, the exposed bone surface is prepared by cells of the osteoblastic lineage which remove unmineralised collagen matrix and deposit a non-collagenous thin layer called “cement-line” that enhances osteoblastic adherence (Zhou et al., 1994) and is later highly mineralised. Why osteoblasts are then recruited exactly where and when osteoclasts have removed bone matrix has prompted a lot of research (Henriksen et al., 2014; Sims and Civitelli, 2014; Sims and Martin, 2014; Kim and Koh, 2019; Durdan et al., 2022). This research has identified a number of osteogenic molecules likely to be released by the osteoclasts including growth factors stored in the bone matrix and solubilized through resorptive activity (Delaisse, 2014). The target cells of these osteogenic factors cannot be the active osteoblasts, that are distant to the resorbing osteoclasts. These signals first reach the cells closest to the osteoclasts, including: 1) bone-lining cells, that have retracted to form a canopy and give the osteoclast access to the bone matrix; and 2) mononucleated bone marrow cells called reversal cells (Andersen et al., 2013). These reversal cells appear elongated with flattened nuclei near the osteoclasts and more cuboidal near the osteoblasts. In both sites these reversal cells were positive for the osteoblastic marker Runx2 and negative for monocytic markers, including osteoclast markers (Andersen et al., 2013). Thus, one could assume that they belong to the osteoblastic lineage and could be osteoblast progenitors.

One of those osteogenic factors involved in the reversal phase is TGF-β, a citokine stored in the bone matrix and released by bone resorption that is known to upregulate osteoclast apoptosis and differentiation of osteoblast precursors while it downregulates the differentiation of mature osteoblasts (Pivonka et al., 2008). In view of these effects, TGF-β could coordinate the transition between resorption and formation at a certain bone site as it causes the osteoblast precursors pool to increase.

Over the last decade great efforts have been made to develop sophisticated bone cell population models (BCPM) that take into account the aforementioned regulatory factors (Komarova et al., 2003; Lemaire et al., 2004; Pivonka et al., 2008; Martínez-Reina and Pivonka, 2019; Martínez-Reina et al., 2021a; Martínez-Reina et al., 2021b; Calvo-Gallego et al., 2022). BCPM are able to simulate the process of BR and take into account all the aforementioned effects, by considering the concentration of the cells and of some biochemical factors involved in that process in a representative volume element (RVE). However, BCPMs are continuous in time and do not account for the unique spatio-temporal features of the BR process, which occurs intermittently, disperse throughout the skeleton and sequentially in the phases of the BMU: activation—resorption—reversal—formation—quiescence. For example, temporal-only BCPMs are not able to distinguish the resorption and formation phases and they model them as simultaneous events. They are not able to simulate either the quiescence period and bone turnover results in a continuous process over time.

Only a few spatio-temporal models of BMU remodelling have been developed which consider different bone cell types and regulatory factors. These models can be subdivided in continuous models based on partial differential equations (PDEs) (Ryser et al., 2009; Buenzli et al., 2011; Kameo et al., 2020) and discrete models based on agent-based (and hybrid) approaches (Buenzli et al., 2012a; Tourolle et al., 2021; Quexada et al., 2022). However, the latter models are quite computationally expensive and particularly, PDE based models require robust numerical integration schemes.

In this work, a previous temporal-only BCPM (Martínez-Reina et al., 2021b) was extended to consider spatio-temporal BMU remodelling. The model proposed here accounts for intermittent activation of BMUs based on damage accumulated in the bone matrix. Osteoclastic and osteoblastic cell populations are activated based on cellular availability, i.e., using cell concentration thresholds. The existence of these thresholds would be justified, at least in the case of bone formation, by the findings of Kristensen et al. These authors highlighted that reversal surfaces show a higher cell density of osteoblast precursors than bone-lining cells on quiescent surfaces. This enrichment was stressed to be obligatory because bone formation is detected only above a certain level of cell density (Kristensen et al., 2014).

These thresholds could be responsible for the occurrence of the formation and resorption cycles. However, the thresholds alone would not be able to separate the resorption and formation events by a reversal period. This separation is probably due to the complex mechanisms triggered by osteoclasts during bone resorption that couple this process to osteoblastogenesis, most notably the release of certain matrix-derived coupling factors. Different molecules have been suggested as potential coupling factors, namely, PDGF, VEGF, BMP-2, IGF-1 and TGF-β (Kim and Koh, 2019). Animal studies using genetically manipulated mice have provided strong evidence that the latter two could be key to linking bone resorption and formation (Tang et al., 2009; Xian et al., 2012). Nevertheless, an inhibitory effect on bone formation has also been attributed to TGF-β, so suggesting that additional coupling factors must promote osteoblast differentiation and bone formation (Durdan et al., 2022). However, this could be explained without the need for other coupling factors, simply by the fact that TGF-β stimulates differentiation of uncommitted osteoblast progenitors, but it inhibits differentiation of osteoblast precursor cells (Janssens et al., 2005). In other words, TGF-β would promote an increase in the concentration of osteoblast precursors, which would eventually lead to the differentiation of active osteoblasts when a certain threshold was exceeded. Since TGF-β also promotes osteoclast apoptosis (Fuller et al., 2000), the transition from resorption to formation, with a lag time needed for the threshold to be exceeded, would be explained by the key role of TGF-β. The previous BCPM considered the concentration of TGF-β among its variables, but with the new model we will show how this factor can regulate the interaction between the cells in the resorption and formation fronts, so leading to the reversal phase addressed before.

The new model was implemented in a Finite Element (FE) code [ABAQUS v2020, Simulia Dassault Systèmes (Dassault Systems, 2022)] and takes into account interactions between neighbouring BMUs. Using this novel model we are able to address a variety of relevant questions related to the bone remodeling process: 1) Are osteoclastic and osteoblastic cell populations activated based on cellular availability? 2) Does spatial segregation of both cell populations depend on the threshold values? 3) Does BMU remodelling regulate the heterogeneous distribution of mineral or bisphophonates within bone matrix?

This paper is organised as follows. In Section 2 we provide a detailed description of the bone remodelling model. In Section 2.1 we focus on the activation and deactivation of BMUs based on cellular availability. In Section 2.2 we present the features of the model that allow to simulate the spatial progression of BMUs. A brief description of the performed simulations as well as the FE model used in them is given in Section 2.3. The *in silico* results are reported and compared with histomorphometric results found in the literature in Section 3, together with a sensitivity analysis of the essential model parameters. The results are discussed in detail in Section 4 and the most relevant conclusions are drawn in Section 5.

## 2 Materials and methods

The proposed model was developed in two steps. First, a previous BCPM describing bone cell interactions (Martínez-Reina et al., 2021b) was modified to take into account the different phases of the BMU: activation, resorption, reversal, formation and quiescence. These phases are not forced to occur, as done in other bone remodelling models (Hazelwood et al., 2001; Martínez-Reina et al., 2009; Martínez-Reina et al., 2014); on the contrary, they emerge as a natural consequence of introducing cellular availability based on cellular concentration thresholds in the model. Secondly, the model, which was originally applied in a RVE, was implemented on a 3D spatial domain with a non-local formulation, i.e., the variables of the model do not only depend on what occurs locally at each integration point, but also on what occurs in the surroundings.

### 2.1 Model of bone cell interactions in BR. Local approach

Following Pivonka et al. (2008), the BR process is described by a set of differential equations of the cell populations. The BCPM considers the RANK–RANKL–OPG signalling pathway, the action of TGF-β, the mechanobiological feedback on bone cells, the effect of bone mineralisation and the accumulation of microstructural damage by fatigue and its repair through BR. The bone cell types considered in the current model are: osteocytes (Ot), osteoblast precursor cells (Ob_p_), active osteoblasts (Ob_a_), osteoclast precursor cells (Oc_p_) and active osteoclasts (Oc_a_). The cell pools of uncommitted osteoblasts (Ob_u_) and osteoclasts (Oc_u_) are assumed constant.

$$\begin{array}{ll} \frac{d\;Ob_{p}}{dt} & =D_{Ob_{u}}\cdot Ob_{u}\cdot \Pi_{\mathrm{act}}^{\mathrm{TGF}-\beta }+P_{Ob_{p}}\cdot Ob_{p}\cdot \Pi_{\mathrm{act}}^{\psi_{bm}}\cdot K_{Ob_{p}}\\ & \;-D_{Ob_{p}}\cdot Ob_{p}\cdot \Pi_{\mathrm{rep}}^{\mathrm{TGF}-\beta }\cdot F_{Ob_{p}} \end{array}$$ (1)

$$\frac{d\;Ob_{a}}{dt}=D_{Ob_{p}}\cdot Ob_{p}\cdot \Pi_{\mathrm{rep}}^{\mathrm{TGF}-\beta }\cdot F_{Ob_{p}}-A_{Ob_{a}}\cdot Ob_{a}$$ (2)

$$\frac{d\;Oc_{p}}{dt}=D_{Oc_{u}}\cdot Oc_{u}\cdot \Pi_{\mathrm{act}}^{\mathrm{RANKL}}-D_{Oc_{p}}\cdot Oc_{p}\cdot \Pi_{\mathrm{act}}^{\mathrm{RANKL}}\cdot F_{Oc_{p}}$$ (3)

$$\frac{d\;Oc_{a}}{dt}=D_{Oc_{p}}\cdot Oc_{p}\cdot \Pi_{\mathrm{act}}^{\mathrm{RANKL}}\cdot F_{Oc_{p}}-A_{Oc_{a}}\cdot Oc_{a}\cdot \Pi_{\mathrm{act}}^{\mathrm{TGF}-\beta }$$ (4)

$$\frac{d\;Ot}{dt}=\eta \frac{df_{bm}}{dt}$$ (5)

where $D_{Ob_{u}}$ , $D_{Ob_{p}}$ , $D_{Oc_{u}}$ and $D_{Oc_{p}}$ are the differentiation rates of Ob_u_, Ob_p_, Oc_u_ and Oc_p_, respectively; while $A_{Ob_{a}}$ and $A_{Oc_{a}}$ are the apoptosis rate of Ob_a_ and Oc_a_, respectively. *η* is the concentration of osteocytes in bone matrix, which is assumed constant as in (Martin et al., 2019), thus leading to proportional variations of osteocytes population and fraction of bone matrix volume per total volume, *f*_*bm*_ (Eq. 5). The second term in the right-hand side of Eq. 1 corresponds to proliferation of osteoblast precursors and $P_{Ob_{p}}$ gives the maximum proliferation rate. The factor $K_{Ob_{p}}$ is introduced following (Buenzli et al., 2012b) and is a saturation function that prevents the proliferation of osteoblast precursors if their population exceeds a certain value $Ob_{p}^{\max}$ .

$$K_{Ob_{p}}=\left\{\begin{array}{cc} 1-\frac{Ob_{p}}{Ob_{p}^{\max}}\; & if\;\;Ob_{p}<Ob_{p}^{\max}\\ 0\; & if\;\;Ob_{p}\geq {Ob_{p}}^{\max} \end{array}\right.$$ (6)

The factors $\Pi_{\mathrm{act}}^{\mathrm{TGF}-\beta }$ and $\Pi_{\mathrm{rep}}^{\mathrm{TGF}-\beta }$ represent activator and repressor functions related to the binding of TGF-β to its receptor. Similarly, $\Pi_{\mathrm{act}}^{\mathrm{RANKL}}$ is the activator function related to the RANK-RANKL binding, while $\Pi_{\mathrm{act}}^{\psi_{bm}}$ is a function of the mechanical stimulus that regulates the anabolic part of the mechanical feedback in the proliferation term.

The mechanical feedback regulation of bone is included in the model following the Mechanostat Theory proposed by Frost (Frost, 2003) and distinguishing three zones (disuse, no net effect in bone mass and overload) as a function of the “Minimally Effective Strains” (MES). The pathologic overload is indirectly considered through the accumulation of microstructural damage. Mechanical disuse is assumed to enhance the production of RANKL on osteoblast precursors depending on the strain energy density (SED) of bone matrix, through the factor $\Pi_{\mathrm{act}}^{\mathrm{RANKL}}$ . Overload is assumed to promote bone formation by proliferation of osteoblast precursors through the activator function $\Pi_{\mathrm{act}}^{\psi_{bm}}$ . The SED, termed here *ψ*_*bm*_, was used as a measure of the mechanical stimulus sensed by bone cells to drive bone adaptation, as traditionally done in the literature (Huiskes et al., 1987; Beaupré et al., 1990). *ψ*_*bm*_ was used here as an alternative to the strains *MES*, used in the Mechanostat Theory (Frost, 2003). In a uniaxial stress state both variables are related through:

$$\psi_{bm}=\frac{1}{2}\;E\cdot {MES}^{2}$$ (7)

*E* being the Young’s modulus. In a general stress-strain state SEDs is given by:

$$\psi_{bm}=\frac{1}{2}\;\sigma_{ij}\;\varepsilon_{ij}$$ (8)

where the Einstein summation convention has been used and *σ*_*ij*_ and *ɛ*_*ij*_ are the components of the stress and strain tensors, respectively. For brevity, only some of the equations of the previously developed model are shown here. The rest are given in Supplementary Section S3.

The main novelty of the present model is the introduction of the concept of cell availability, a threshold based phenomenon whereby we assume that the differentiation of precursors into mature cells is activated only when the population of the former reaches a certain upper threshold and continues until that population falls below a lower threshold. Thus, two binary variables $F_{Ob_{p}}$ and $F_{Oc_{p}}$ are defined to activate (F_X_ = 1, with X = Oc_p_ or Ob_p_) or deactivate (F_X_ = 0) the differentiation of osteoblasts and osteoclast precursor cells, respectively.

$$\text{if}\;Ob_{p}>Ob_{p}^{up,th}\to F_{Ob_{p}}=1\;\text{until}\;Ob_{p}<Ob_{p}^{\mathrm{low,th}}\to F_{Ob_{p}}=0$$ (9a)

$$\text{if}\;Oc_{p}>Oc_{p}^{up,th}\to F_{Oc_{p}}=1\;\text{until}\;Oc_{p}<Oc_{p}^{\mathrm{low,th}}\to F_{Oc_{p}}=0$$ (9b)

We note that in Eq. 6 $Ob_{p}^{\max}>Ob_{p}^{up,th}$ . Therefore, following Eqs 9a, 9b, precursor cells accumulate until their population reaches the upper threshold, when they begin to differentiate into their respective mature (and active) forms. Then, the precursor cell pool drops to the lower threshold, when the concentration is not sufficient to ensure the continuity of the process and at that moment differentiation stops. At this point, the concentration of precursor cells can rise again if the necessary environmental factors are met and a new remodelling cycle can start if the upper threshold is reached again.

**FIGURE 1:**
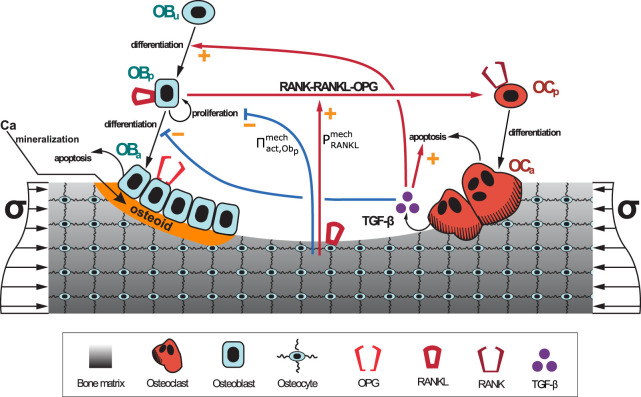
Schematic representation of the BCPM developed in (Martínez-Reina et al., 2021b): bone cell differentiation stages along with biochemical and biomechanical interactions are presented. The mineralisation of osteoid is shown in orange.

The action of mature osteoclasts and osteoblasts in the BMU is reflected by temporary or permanent changes in porosity. Bone matrix fraction is defined as the volume of bone matrix, *V*_*b*_, per total volume of the bone sample, *V*_*T*_, expressed as a percentage, i.e., $f_{bm}\left(\%\right)=\frac{V_{b}}{V_{T}}\cdot 100$ . Its variation is obtained through the balance between resorbed and formed tissue:

$$\frac{df_{bm}}{dt}=-k_{\mathrm{res}}\cdot Oc_{a}+k_{\mathrm{form}}\cdot Ob_{a}$$ (10)

where *k*_*res*_ and *k*_*form*_ are, respectively, the rates of bone resorption and osteoid formation. Figure 1 shows a schematic representation of the BCPM model (Martínez-Reina et al., 2021b) on which the current spatio-temporal model is based. The values of the constants of the BCPM are given in Supplementary Table S1.

The mathematical framework described above can be applied in a bone RVE to reproduce the different phases of an isolated BMU acting locally within the RVE. However, if the RVE is large enough to hold several BMUs, modelling the discrete activation of a single BMU is no longer meaningful and, more importantly, the interactions between those BMUs cannot be modelled in the sense addressed in this work. For this reason, a 3D domain must be considered to account for these interactions. In particular, a 3D FE mesh is proposed in a non-local approach, with each element being small enough to hold at most one BMU. In this way, the movement of the BMU can also be modelled by changing the element that contains the BMU at each time.

### 2.2 3D spatio-temporal bone remodelling. Non-local approach

In a FE mesh, the conditions for activation, deactivation and progression of a BMU should be determined. In the local approach, addressed in the previous section, the only condition to be fulfilled in order to activate a BMU was that the concentration of Oc_p_ must be higher than a certain threshold for them to differentiate into Oc_a_. In the following, an element [or better an integration point (IP)]^1^ where the differentiation of precursor cells into mature cells is taking place will be called “active” and this includes active resorption and active formation, since each process is evaluated separately. For the non-local approach proposed in this work, other conditions are imposed in order to prevent the activation of a certain process (resorption or formation) if the same process is active in a nearby element. We hypothesize that a new BMU will not be activated if an existing BMU is progressing in the neighbourhood. Instead, the metabolic cost of activating a new BMU will be employed in “feeding” the existing BMU, with the recruitment of more progenitor cells to the resorbing and formation sites, respectively. To do so, the model must take into account what is occurring in the vicinity of a given IP and not only the local events.

The neighbourhood is defined following the algorithm proposed by Calvo-Gallego et al. (2021). For a given IP, *p*, a neighbourhood of IPs, *NBH* ^*p*^, is defined by the IPs contained in a sphere of radius *R* centered at *p*:

$$NBH^{p}=\left\{i\in \left(\text{IPs of FE mesh}\right),\;i=1,\ldots ,N_{p};\;\text{such that}\;\;\|r_{i}-r_{p}\|\leq R\right\}$$ (11)

where **r**_*i*_ denotes the position of IP *i*. Next, the resorption and formation fronts of the BMU are distinguished. To this end, two state variables are defined at each IP: resorption state, *RS*, and formation state, *FS*, so that:

$$RS=\left\{\begin{array}{cc} 1\; & \text{if this IP is the resorption front}\;\Rightarrow \;F_{Oc_{p}}=1\\ 2\; & \text{resorption is active but this IP is not the front}\;\Rightarrow \;F_{Oc_{p}}=1\\ 0\; & \text{resorption is inactive at this IP}\;\Rightarrow \;F_{Oc_{p}}=0 \end{array}\right.$$ (12)

$$FS=\left\{\begin{array}{cc} 1\; & \text{if this IP is the formation front}\;\Rightarrow \;F_{Ob_{p}}=1\\ 2\; & \text{formation is active but this IP is not the front}\;\Rightarrow \;F_{Ob_{p}}=1\\ 0\; & \text{formation is inactive at this IP}\;\Rightarrow \;F_{Ob_{p}}=0 \end{array}\right.$$ (13)

The procedure to establish if one process (either resorption or formation) is activated or deactivated at a certain IP at the instant *t*_*a*_ is described next. It is important to note that resorption and formation are treated separately. For this reason, in the following procedure, the state variables are designated as *XS*, where *X* can stand either for *R* or *F*. The coupling of both processes and the correct sequence (resorption, reversal, formation) is not enforced, but emerges as a model output.

**FIGURE 2:**
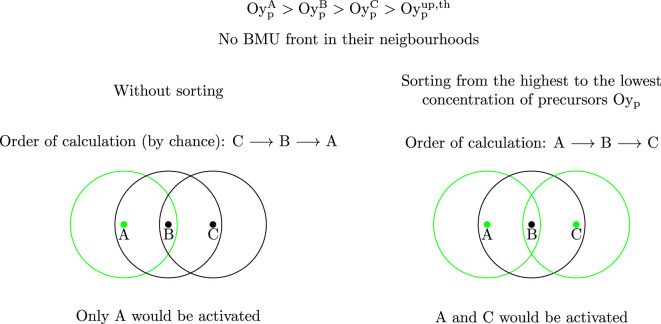
Rationale for sorting the IPs from the highest to the lowest concentration of precursors. The IPs which would be activated are represented in green. If a gradient of precursors concentration existed $\left(Oy_{p}^{A}>Oy_{p}^{B}>Oy_{p}^{C}>Oy_{p}^{up,th}\right)$ , the IPs to be activated would depend on the order in which the FE software analyses the IPs. If this order were by chance C, B, A (left), only the IP A would be activated. C is analysed first, but the algorithm detects that B (which is inside *NBH* ^*C*^) hosts the maximum of Oy_p_ within *NBH* ^*C*^ and prevents the activation at C. Next, B is analysed, but the activation is here prevented by the maximum of Oy_p_ within *NBH* ^*B*^, which occurs at A. Therefore, only A is activated. By sorting the IPs by Oy_p_, i.e., A,B,C (right), this randomness is avoided and both A and C would be activated, since C ∉*NBH* ^*A*^.

1. First, the IPs of the mesh are ordered from the highest to the lowest concentration of precursors Oy_p_, where y stands for b or c depending on the process being activated. In the next steps the IPs will be analysed in this order. The rationale for this is explained in detail in Figure 2.
2. For each IP *j* it is checked if the neighbourhood *NBH* ^*j*^ contains another IP with an already active front (i.e., if *XS* = 1 for *t*_*a*−1_).^2^ If not, the IP *j* is included in the subset of candidates for activation, *C*. If there is another IP in *NBH* ^*j*^ with an active process, activation must be prevented and this IP *j* is excluded from the subset of candidates, *C*.

Next, the following conditions are checked for the subset *C*.

3. The concentration of precursor cells Oy_p_ at IP *j* is the highest in *NBH* ^*j*^.
4. The concentration of precursor cells Oy_p_ exceeds the corresponding upper threshold, i.e., $Oy_{p}>Oy_{p}^{up,th}$ .

These are the conditions for the activation of a new process. Once activated, the progression of each process along the mesh is subjected to the fulfillment of the following criteria, otherwise the front will not move.

5. The concentration of precursor cells (osteoclasts or osteoblasts) of the destination IP must be above the corresponding upper differentiation threshold.
6. The origin IP is the current front. The destination IP must be inside the neighbourhood of the origin IP.
7. The IP with the highest concentration of precursor cells inside that neighbourhood is the first destination IP. Consequently, this concentration must also be higher than that at the origin.
8. At this point the BMU can branch and another destination IP can become a new front if the three previous conditions are fulfilled and if this second destination IP is not inside the neighbourhood of the first destination IP. Multiple branches could appear, but the conditions are very hard to meet in this case and no multiple branches have been observed in our simulations.

If these criteria are fulfilled, the destination IPs become the new fronts (*XS* = 1) and the origin IP becomes a simple active point *XS* = 2. It must be noted that a smooth progression of the BMU is not guaranteed. Indeed, the BMU could theoretically leap one or more IPs if the radius of the neighbourhood spans more than 2 elements and this would be a limitation of the model which can be avoided by choosing a sufficiently small radius R.

9. A certain process is deactivated at an IP if the concentration of precursor cells (osteoclasts or osteoblasts) falls below the lower differentiation threshold. In this case *XS* changes from 1 or 2 to 0.

The activation of a BMU starts when the first osteoclasts are differentiated from their precursors (i.e., when the upper threshold $Oc_{p}^{up,th}$ is exceeded and $F_{Oc_{p}}=1$ ) at an IP of a neighbourhood where there was no other existing BMU. At this IP *RS* becomes equal to 1.

To summarize the algorithm, each process of the BMU (resorption or formation) is activated at the point where the concentration of precursors is the highest to proceed with the differentiation of precursors into active cells. This differentiation will proceed along the gradient of precursors concentration until it is deactivated, when the concentration of precursors is below the lower threshold. In view of Eq. 3, an increase of RANKL production will lead to a rise in the concentration of osteoclast precursors. One possible reason for that RANKL increase is the accumulation of microstructural damage (Martínez-Reina et al., 2021b) through the factor $\Pi_{\mathrm{act}}^{\mathrm{dam}}$ (see Supplementary Section S2.3 for more details). Thus, one of the reasons for the BMU resorption front to move in this model is to repair highly damaged areas, in accordance with the theory of targeted bone remodelling (Parfitt, 2002). On the other hand, the concentration of osteoblast precursors will increase in overloaded areas, as the proliferation term in Eq. 1 is upregulated by mechanotransduction through the factor $\Pi_{\mathrm{act}}^{\psi_{bm}}$ . Ob_p_ will also increase in areas where resorption has recently taken place, as osteoclasts release TGF-β from bone matrix and this upregulates the differentiation of uncommitted osteoblasts into osteoblast precursor cells through $\Pi_{\mathrm{act}}^{\mathrm{TGF-\beta}}$ (see Eq. 1). Hence, the BMU formation front will follow the path previously set by osteoclasts until formation is deactivated, so allowing the different phases in sequential order: activation, resorption, reversal, formation and quiescence.

The role of TGF-β in this sequence is paramount as will be shown later in the results. Its concentration in the bone compartment is governed by the following differential equation in the original model (Pivonka et al., 2008):

$$\frac{d\;\left[TGF-\beta \right]}{dt}=P^{\text{TGF}-\beta }+\alpha_{\text{TGF}-\beta }\cdot k_{\mathrm{res}}\cdot Oc_{a}+{\tilde{D}}_{\text{TGF}-\beta }\cdot \left[TGF-\beta \right]$$ (14)

where *P* ^TGF−β^ is the external production of TGF-β. The second term on the right-hand side represents the release of TGF-β from bone matrix through bone resorption, with *α*_TGF−β_ being the concentration of TGF−β in bone matrix. Finally, ${\tilde{D}}_{\text{TGF}-\beta }$ is the TGF-β degradation rate. In temporal-only BCPMs, stationarity of Eq. 14 is assumed in order to compute the TGF-β concentration in the RVE.^3^ However, assuming stationarity in the current model would neglect the effect that the release, action and degradation of TGF-β has on the coupling of the BMU processes. Therefore, the differential Eq. 14 is solved in the current model. We note that Buenzli et al. (2012a) also pointed out that the distribution of TGF-β from the BMU cutting cone to the closing cone has a significant effect on bone cell density distribution and separation of cell populations.

### 2.3 FE model

**FIGURE 3:**
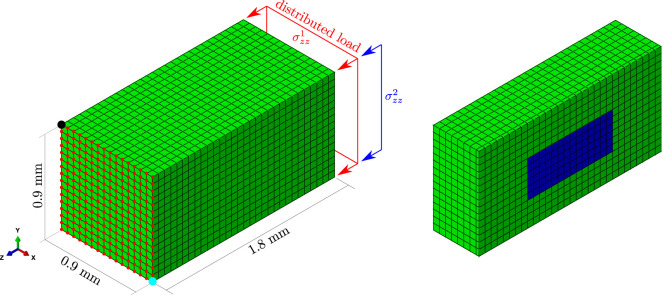
FE mesh used in the simulations. Cubic elements of side 60 *μ*m and type C3D8R from the ABAQUS Element Library (linear 8-noded hexahedra with reduced integration, 1 integration point) were used. The piece of bone was subjected to uniaxial compression, uniaxial tension or compression plus bending, by applying a distributed load in one face (and also in one edge in the case of compression plus bending) and by preventing certain displacements in the opposite face. On the right-hand side, the model is cut to show in blue a YZ section of the domain under study, which spans a depth of 6 elements in the X direction and is centered along this direction.

The new spatio-temporal BCPM model was tested in a 3D domain (see Figure 3). It was developed in ABAQUS and consists of a parallelepiped of dimensions 0.9 × 0.9 × 1.8 mm^3^ (see Figure 3). It was meshed with 6,750 eight-noded hexahedral isoparametric elements, with reduced integration (one integration point per element, named C3D8R in the ABAQUS Element Library). All hexahedra are regular of side 60 *μ*m. The choice of this size was based on the fact that a BMU progresses at a rate between 20 *μ*m/day (Parfitt, 2002) and 40 *μ*m/day (Martin et al., 2015). Besides, the model corresponds to a piece of trabecular bone which is analysed at the mesoscale, i.e., from a Continuum Mechanics point of view and without considering its microstructure, though accounting for the spatial variation of *f*_*bm*_ across the trabecular structure at the mesoscale. For that purpose, an element size of 60 *μ*m is also adequate.

Three load cases were analysed: uniaxial compression ( $\sigma_{zz}^{1}=-0.5$ MPa), uniaxial tension ( $\sigma_{zz}^{1}=+0.5$ MPa) and compression plus bending ( $\sigma_{zz}^{1}=-0.5$ MPa and $\sigma_{zz}^{2}=-1.33$ N/mm, so that the longitudinal stresses ranged from −0.2 to −0.8 MPa). The boundary conditions were chosen to ensure a uniform uniaxial stress state in the first two cases. More precisely, the Z displacement of all nodes of one face (red dots, see Figure 3 left); the Z and Y displacements in one corner (blue dot) and the Z and X displacements in the opposite corner (black dot) were restrained to prevent the rigid-body motions. The same boundary conditions were applied to the compression plus bending case.

A subregion of this model (blue in Figure 3 right), far enough from boundary conditions and concentrated loads, was selected to evaluate the results. This subregion is a parallelepiped composed of 504 elements (6 × 6 × 14) and placed in the core of the model. This subregion is hereinafter referred to as the domain under study. Two cases have been simulated: a homeostasis situation and a model including a highly damaged region.

#### 2.3.1 Homeostasis

The first objective was to investigate whether a homeostasis situation can be reached in the FE mesh under the loading conditions specified above. The initial conditions imposed to each element were obtained as follows. First, the previous continuous BCPM model (Martínez-Reina et al., 2021b) was run in a RVE under 0.5 MPa uniaxial compression, in a similar way to what Smit and Burger did in (Smit and Burger, 2010). This load was applied during a period of time long enough to reach an equilibrium or homeostatic state. The values of all the variables at this state were imposed as uniform initial conditions except for *f*_*bm*_, damage and Oc_p_, which were perturbed. More precisely, a value within a certain range was randomly assigned to the elements: [2.5 ⋅ 10^–6^, 3.5 ⋅ 10^–6^] for the initial damage, [0.5 ⋅ 10^–4^, 1.5 ⋅ 10^–4^] pM for the initial concentration of Oc_p_ and [10, 80]% for the initial *f*_*bm*_, with an average value around 40%. As stated before, the FE model represents a piece of trabecular bone analysed at the mesoscale. Each finite element acts as the RVE in the current analysis, but the distribution of *f*_*bm*_ across the mesh must account for the spatial variation of bone volume fraction within the trabecular structure at the mesoscale. For this reason, the value of *f*_*bm*_ in individual elements can be out of the normal range for trabecular bone, though in a larger scale (macroscopic, for example, the whole FE model or a few tens of elements) its average does correspond to trabecular bone. To analyse the effect of the initial distribution of *f*_*bm*_, the latter results will be compared with those obtained for an initial *f*_*bm*_ ∈ [27, 29]%.

The aim of those perturbations was to start from a more realistic situation than a uniform distribution of all the variables. Three sources of deviation from homeostasis are implicit in this procedure: 1) the equilibrium in the continuous BCPM model does not necessarily coincide with that in a 3D FE mesh, because in the latter we implement restrictions for the activation, movement and deactivation of BMUs; 2) the initial conditions are not uniform due to the random assignment of *f*_*bm*_, damage and Oc_p_; 3) a value *f*_*bm*_ = 40% may not correspond to the porosity in equilibrium with 0.5 MPa, but this will test the model’s ability to reach the homeostatic situation by changing *f*_*bm*_ with time.

The second objective was to evaluate if the different phases of the BMU cycle and their lengths appeared correctly, as well as the BMU activation frequency. The average length of resorption and formation periods are defined as the mean time required to complete bone resorption and formation, respectively, at a certain point (IP in this study). The average length of reversal period is defined as the mean time elapsed between the end of resorption and the beginning of formation at a certain point. Finally, the quiescence period is defined as the mean time elapsed between the end of formation and the beginning of the next resorption cycle, i.e., the period when no remodelling takes place at the considered point.

The activation frequency is defined in two different ways in the literature, based on 3D or 2D measurements (Martin, 1994). The former, given by Frost in 1964 (Frost, 1964), defined the activation frequency as the number of BMUs activated per unit volume and unit time. From this perspective, the observer would follow the BMU progressing from the activation of the first osteoclasts until osteoblast formation ceases completely. The 2D definition arises from histomorphometric studies, in which histologic sections are used to characterize the process. Here, one activation is counted after the appearance of a BMU in the section under study. Histology-based techniques are still the gold standard for analysing bone microstructure. Although many promising methods are arising to measure the 3D BMU parameters, no study reporting an experimentally assessed activation frequency in 3D has been found by the authors. Martin (1984) and Hernandez et al. (1999) provided equations to relate the activation frequency in 2D and 3D, although they already warned that the equations depend on parameters which are difficult to estimate and are not measured in detail. Therefore, we provide here the 3D activation frequency, which can be easily calculated in a FE model, although this cannot be compared with 2D experimental results. For comparison, we have also estimated the 2D activation frequency as the inverse of the total BMU cycle time (see Eq. 16).

Finally, the influence of TGF-β in the remodelling process was also studied. As discussed above, TGF-β is a citokine that could play a key role in coordinating resorption and formation at a certain bone site. To evaluate its effect, a special case simulating the absence of TGF-β was analysed, i.e., if no TGF-β was released from bone matrix through resorption or equivalently *α*_*TGF*−β_ = 0 (see Eq. 14).

#### 2.3.2 Considering targeted bone remodelling due to microdamage

**FIGURE 4:**
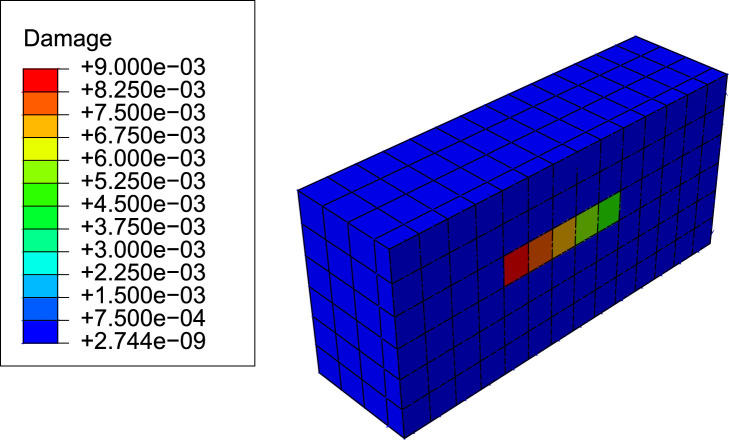
Initial damage distribution for the targeted bone remodelling simulations (view cut by a middle plane of the subregion of the model where results were evaluated).

The following simulations aim to demonstrate the ability of the current model to simulate the repairing of microstructural damage as hypothesized in targeted bone remodelling. A highly damaged region was considered by setting a line of five elements along the *z*-direction in the core of the model with a damage level much higher than the rest of the domain and a gradient of damage as shown in Figure 4. Damage, *d*, is a variable in the range [0, 1] that is related to the degradation of stiffness, so that *d* = 0 stands for an intact (undamaged) element, while *d* = 1 stands for local failure (see Supplementary Section S4). Pattin et al. (1996) showed experimentally, in fatigue tests performed in cortical bone samples, that *d* ∼ 0.01 represents indeed a high damage level, as fatigue failure can occur a few cycles after that value had been reached, depending on the applied load. The objective was to evaluate whether a BMU is activated in the element with the highest damage and that it progresses along the damage gradient in order to repair, or at least to reduce, the level of damage.

## 3 Results

### 3.1 Calibration of thresholds

The thresholds in Eq. 9 were calibrated to obtain a BMU activation frequency and lengths of the BMU phases in accordance with the literature. For this purpose, the thresholds were varied in the following ranges: $Ob_{p}^{up,th}\in \left[0.04,\;0.02\right]$ , $Ob_{p}^{\mathrm{low,th}}\in \left[0.0025,\;0.0005\right]$ , $Oc_{p}^{up,th}\in \left[0.025,\;0.005\right]$ and $Oc_{p}^{\mathrm{low,th}}\in \left[0.0025,\;0.0005\right]$ . These ranges were divided in 3 equally spaced subranges and all the combinations of the resulting 4 points of each threshold were evaluated. Subsequently, a local gradient search was performed starting from the best combination, being the total difference with the lengths of Table 2 the objective function to be minimised in this search. The optimum is shown in Table 1 and this was used for the nominal values of the thresholds. A sensitivity analysis of the thresholds was performed around those nominal values to evaluate their effect on the length of the phases.

**TABLE 1:** Nominal values of the thresholds.

$Ob_{p}^{up,th}$	$Ob_{p}^{\mathrm{low,th}}$	$Oc_{p}^{up,th}$	$Oc_{p}^{\mathrm{low,th}}$
2.8 ⋅ 10^–2^	9.0 ⋅ 10^–4^	1.5 ⋅ 10^–2^	8.0 ⋅ 10^–4^

### 3.2 Homeostasis

To check whether homeostasis was reached after the initial perturbation, the evolution of *f*_*bm*_ was analysed in the domain under study for the compression load case. First, the average of *f*_*bm*_ was calculated for the elements of the domain under study to yield $f_{bm}^{\mathrm{aver}}\left(t\right)$ . The temporal evolution of $f_{bm}^{\mathrm{aver}}\left(t\right)$ showed an initial adaptation to the applied load, which was low for the initial distribution of *f*_*bm*_, thus resulting in a decrease of bone mass. During this phase of adaptation very pronounced fluctuations were observed as a consequence of the numerous remodelling events occurring within the domain. Thus, a moving average filter was applied to the temporal evolution of $f_{bm}^{\mathrm{aver}}\left(t\right)$ to give:

$$f_{bm}^{mv}\left(t\right)=\overset{t+n/2}{\underset{t-n/2}{\sum }}f_{bm}^{\mathrm{aver}}\left(t\right)$$ (15)

where *n* is the number of time points considered for the average (or time window). Note that *t* + *n*/2 cannot exceed the simulation time (*t*_*max*_) and *t* − *n*/2 cannot be less than 0 and therefore, the window must be truncated at those endpoints.

**FIGURE 5:**
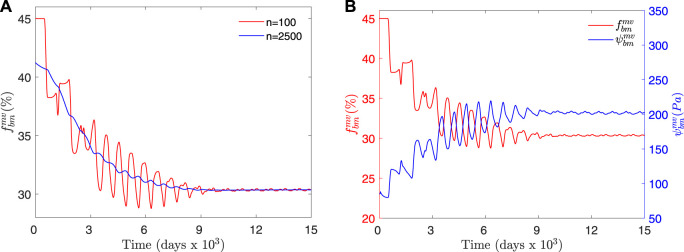
**(A)** Temporal evolution of $f_{bm}^{mv}$ (%) for two different time windows of the moving average. First, the average *f*_*bm*_ is adapted to the applied load and then stabilized to reach a homeostatic situation with small oscillations. **(B)** Temporal evolution of $f_{bm}^{mv}$ (%) and $\psi_{bm}^{mv}$ (Pa), both averaged for a time window *n* = 100. This evolution corresponds to the compression load case with an initial random distribution of *f*_*bm*_ ∈ [10, 80]%.

**FIGURE 6:**
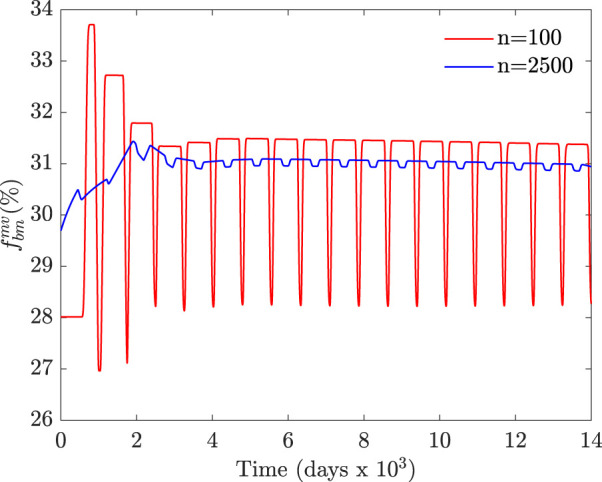
Temporal evolution of $f_{bm}^{mv}$ (%) for two different time windows of the moving average. This evolution corresponds to the compression load case with an initial random distribution of *f*_*bm*_ ∈ [27, 29]%.

The evolution of *ψ*_*bm*_ was also obtained in the domain under study, to analyse the relationship between *f*_*bm*_ and the mechanical stimulus. $\psi_{bm}^{\mathrm{aver}}$ and $\psi_{bm}^{mv}$ were calculated through spatial and temporal averaging of *ψ*_*bm*_, analogously to $f_{bm}^{\mathrm{aver}}$ and $f_{bm}^{mv}$ . Figure 5A shows the temporal evolution of $f_{bm}^{mv}$ after the filter was applied for two different time windows. Figure 5B compares $f_{bm}^{mv}\left(t\right)$ and $\psi_{bm}^{mv}\left(t\right)$ . Figure 5 corresponds to the compression load case with an initial random distribution of *f*_*bm*_ ∈ [10, 80]%. The evolution of $f_{bm}^{mv}\left(t\right)$ when initial *f*_*bm*_ ∈ [27, 29]% is shown in Figure 6 for comparison.

**FIGURE 7:**
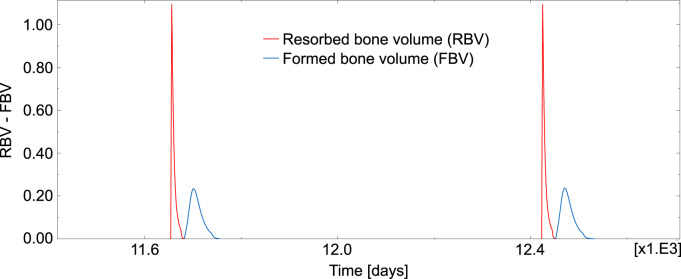
Evolution of RBV and FBV for one element inside the domain under study.

Figure 7 represents the temporal evolution of resorbed bone volume and formed bone volume per unit volume and unit time (i.e., RBV = *k*_*res*_ ⋅Oc_a_ and FBV = *k*_*form*_ ⋅Ob_a_), for an element inside the domain under study.

Several activations of BMUs took place in the domain under study, though not simultaneously. The average length of the resorption, inversion (or reversal), formation and quiescence phases was calculated from the results of all the elements inside the domain under study. The first phase corresponds to a transient period during which the bone volume fraction is adapting to the applied load and must be ruled out to focus on the homeostatic situation, which was assumed to be reached around day 10,000 (see Figure 5). The average lengths of the phases were calculated from day 13,000 until the end of the simulation (day 15,000). These averages are given in Table 2 and compared with experimental data from the literature. The activation frequency is calculated in histomorphometric studies from the average length of the BMU phases (Eriksen et al., 1990; Steiniche et al., 1994) and represents the number of BMUs passing through a given bone site per year:

$$f_{\mathrm{act,hist}}\left(\frac{BMUs}{year}\right)=\frac{365}{T_{R}+T_{I}+T_{F}+T_{Q}}$$ (16)

where *T*_*R*_, *T*_*I*_, *T*_*F*_ and *T*_*Q*_ are the average resorption, inversion (reversal), formation and quiescence periods. The values obtained for this frequency are given and compared with the literature in Table 2.

**TABLE 2:** Duration in days of the different phases of the BMU cycle and activation frequencies obtained for the three load cases. Comparison of the model results and the existing literature.

Variable	Model			Literature
	Compression	Tension	Compression + bending	
*T*_*R*_ (days)	21.9	21.1	21.9	24 Hazelwood et al. (2001)
*T*_*I*_ (days)	8.3	8.8	8.2	8 Hazelwood et al. (2001)
*T*_*F*_ (days)	65.5	65.4	65.5	64 Hazelwood et al. (2001)
*T*_*Q*_ (days)	595	562	595	597 Steiniche et al. (1994)
$f_{\mathrm{act,hist}}\;\left(\frac{BMUs}{year}\right)$	0.53	0.56	0.53	0.52 Steiniche et al. (1994)
$f_{\mathrm{act,3D}}\;\left(\frac{BMUs}{year\cdot mm^{3}}\right)$	1.55	1.58	1.55	1–2 (*f*_*act,2D*_) Frost (1964)

The 3D BMU activation frequency, *f*_*act,3D*_, was calculated by counting the number of activations of BMUs occurring within the domain under study per unit time and unit volume. This 3D activation frequency can be converted into a 2D activation frequency following Martin (1984) who related both through:

$$f_{\mathrm{act,3D}}=k\cdot f_{\mathrm{act,2D}}$$ (17)

with *k* being a length parameter. Later, Hernandez et al. (1999) used *k* = 1, so identifying the 2D and 3D frequencies. If this assumption is accepted, the estimated values of *f*_*act,2D*_ would be in the range given by Frost (1964) (see Table 2).

The results of the sensitivity analysis are presented in Table 3 for the case of compression. The influence of the thresholds and *R* (the radius of the sphere defining the neighbourhood) was studied.

**TABLE 3:** Sensitivity analysis of $Ob_{p}^{\mathrm{low,th}}$ , $Oc_{p}^{\mathrm{low,th}}$ , $Ob_{p}^{up,th}$ , $Oc_{p}^{up,th}$ and *R*.

$Ob_{p}^{\mathrm{low,th}}$	*f*_*act,3D*_	*T*_*R*_	*T*_*I*_	*T*_*F*_	*T*_*Q*_
1.08 ⋅ 10^–3^	1.64	21.7	7.9	63.8	590
0.99 ⋅ 10^–3^	1.62	21.8	8.1	64.5	593
0.90 ⋅ 10^–3^	1.55	21.9	8.3	65.5	595
0.81 ⋅ 10^–3^	1.59	22.0	8.4	65.7	598
0.72 ⋅ 10^–3^	1.50	22.1	8.8	67.0	601

$Oc_{p}^{\mathrm{low,th}}$	*f*_*act,3D*_	*T*_*R*_	*T*_*I*_	*T*_*F*_	*T*_*Q*_
9.60 ⋅ 10^–4^	1.71	20.4	9.9	64.6	588
8.80 ⋅ 10^–4^	1.63	21.1	9.1	64.9	591
8.00 ⋅ 10^–4^	1.55	21.9	8.3	65.5	595
7.20 ⋅ 10^–4^	1.59	22.2	7.9	65.8	598
6.40 ⋅ 10^–4^	1.43	23.0	7.3	66.3	601

$Ob_{p}^{up,th}$	*f*_*act,3D*_	*T*_*R*_	*T*_*I*_	*T*_*F*_	*T*_*Q*_
3.36 ⋅ 10^–2^	1.41	21.7	288.0	105.0	443
3.08 ⋅ 10^–2^	1.57	21.7	60.0	59.0	536
2.80 ⋅ 10^–2^	1.55	21.9	8.3	65.5	595
2.52 ⋅ 10^–2^	1.64	21.9	4.5	68.8	598
2.24 ⋅ 10^–2^	1.38	22.0	3.0	71.1	599

$Oc_{p}^{up,th}$	*f*_*act,3D*_	*T*_*R*_	*T*_*I*_	*T*_*F*_	*T*_*Q*_
1.80 ⋅ 10^–2^	1.21	23.6	3.55	81.73	717
1.65 ⋅ 10^–2^	1.40	22.9	4.0	70.6	661
1.50 ⋅ 10^–2^	1.55	21.9	8.3	65.5	595
1.35 ⋅ 10^–2^	1.83	20.2	63.7	55.6	461
1.20 ⋅ 10^–2^	1.63	19.9	251.0	75.2	306

*R*	*f*_*act,3D*_	*T*_*R*_	*T*_*I*_	*T*_*F*_	*T*_*Q*_
1.5	0.62	22.0	8.8	65.0	594
1.2	1.27	21.9	8.4	65.3	595
0.9	1.55	21.9	8.3	65.5	595
0.6	2.14	21.9	8.1	65.8	595
0.3	2.46	21.8	8.8	65.6	594

*T*_*R*_, *T*_*I*_, *T*_*F*_ and *T*_*Q*_ are the average resorption, reversal, formation and quiescence times in days, respectively. *f*_*act,3D*_ is the 3D activation frequency in BMUs/year/mm^3^. The nominal values are highlighted in grey.

**FIGURE 8:**
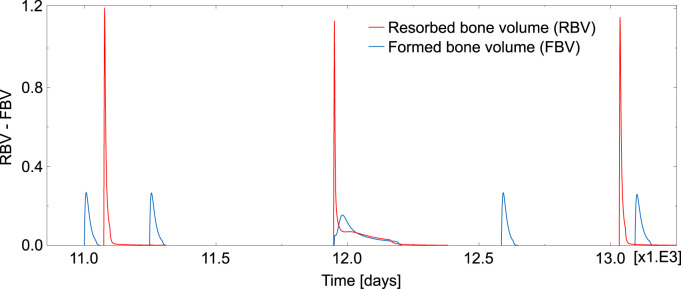
Temporal evolution of RBV and FBV obtained in one element in the case of absence of TGF-β. This time window was chosen for it is representative of the uncoordinated behaviour observed in the absence of TGF-β, with cycles where formation precedes resorption, isolated cycles, overlapping cycles and also normal cycles.

Regarding the simulation of the case of absence of TGF-β, the evolution of the resorbed bone volume per unit time and unit volume (RBV) and the corresponding formed bone volume (FBV) is plotted in Figure 8 for an element inside the domain under study. Compared to Figure 7, where the cycles had a reasonable shape and followed the normal sequence resorption—inversion—formation—quiescence, now the BMU cycles appeared totally uncoupled. Different rare events can be seen in Figure 8: several formation cycles taking place (before or after a resorption cycle), resorption and formation occurring simultaneously and cycles with highly variable resorption and formation lengths.

To quantify the occurrence of these rare events, three types of resorption/formation cycles have been defined.

• A BMU cycle (or remodelling event) is regarded normal if a resorption cycle is followed by a formation cycle in less than $T_{I}^{\max}=20$ days, the maximum admissible inversion time.
• An isolated resorption event is defined as a resorption cycle not followed by a formation cycle in less than $T_{I}^{\max}$ days, either because it takes longer for the formation cycle to start or because the resorption cycle is followed by another resorption cycle. An isolated formation event is defined analogously, either because the previous cycle was also a formation one or because the preceding resorption cycle occurred more than $T_{I}^{\max}$ days ago.
• Resorption/formation cycles are denoted as overlapping cycles when resorption and formation take place at the same time.

All these cycles have been counted in each element of the domain under study in the normal case and in the case of absence of TGF-β. The results are presented in Table 4 for the three load cases. The temporal evolution of *f*_*bm*_ in the absence of TGF-β (not shown) is similar to that of Figure 5A, though with more irregular oscillations.

**TABLE 4:** Count of normal cycles and rare events for the three load cases, in the normal simulations and in the cases where no TGF-β is released from bone matrix by resorption.

Cycle type	Compression		Tension		Compres. + bending	
	Normal	No TGF-β	Normal	No TGF-β	Normal	No TGF-β
Normal	94.2%	0%	95.6%	0.03%	93.4%	0.06%
Isolated Resorption	2.89%	32.1%	1.95%	0.05%	3.14%	32.4%
Isolated Formation	2.89%	61.8%	2.45%	47.9%	3.45%	61.5%
Overlapping	0%	6.05%	0%	52.0%	0%	6.08%

### 3.3 Simulations of targeted bone remodelling due to microdamage

**FIGURE 9:**
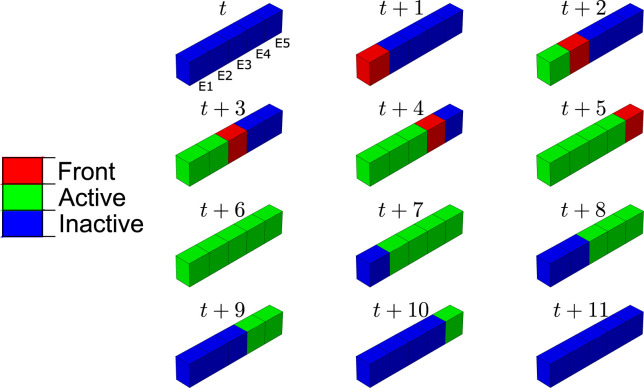
Progression of the BMU resorption front along the damage path and elements where the differentiation from precursor cells to mature cells is active, at day *t* and subsequent days. The elements are named in the first frame from E1 to E5 in decreasing order of damage (see Figure 4). The compression load was applied in this case.

In order to analyse how the BMU progresses along bone matrix repairing the damaged tissue, we have studied the BMU front and those elements where the differentiation from precursor cells to mature cells is active. In Figure 9, the line of highly damaged elements are shown at different times during the resorption cycle: in blue, those where differentiation is not active; in red, the BMU front, with active differentiation; in green, those elements with active differentiation but which are no longer the BMU front. It can be noted that the BMU was activated at the element with the highest damage (recall Figure 4). Later, the BMU front progressed along the damage gradient, towards the next elements with high damage. Differentiation of mature osteoclasts keeps active in those elements through which the BMU front runs, so resorbing bone and repairing damage until the precursors concentration falls below the lower threshold, when they become inactive.

**FIGURE 10:**
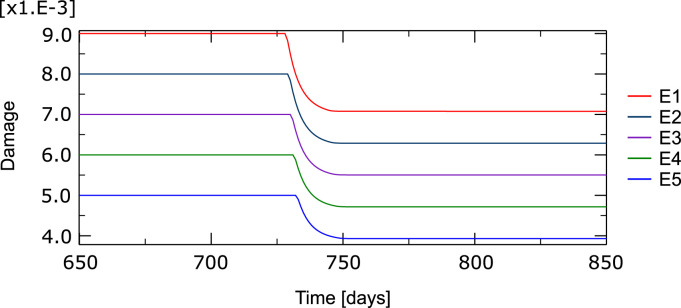
Temporal evolution of the damage variable in the five elements with a high level of initial damage (E1 to E5, see Figure 9). Around day 730 the BMU is activated, first in the most damaged element, and advance through these particular elements partially repairing the tissue through resorption.

The evolution of the damage variable over time in the compression load case can be seen for the damaged elements in Figure 10. It can be noted how a fraction of damage is repaired in the first element when the BMU is activated and how the repair process progresses along the line of damaged elements. Between 20% and 25% of the original damage is repaired.

## 4 Discussion

We have presented in this paper a new spatio-temporal model of bone remodelling based on BMUs. In contrast to the previously developed temporal-only model (Martínez-Reina et al., 2021b), which is based on a set of continuous differential equation, the model proposed here includes additional binary functions that activate or deactivate the differentiation of precursors into mature active cells. This leads to an intermittent activation of the resorption and formation processes that simulate spatio-temporal BMU remodelling in a physiological meaningful way. Indeed, the well-known sequence of the BMU: resorption—reversal—formation—quiescence emerged from the simulations, by simply including some thresholds in the differentiation processes. We note that these thresholds for Ob_p_ and Oc_p_ account for the availability of bone precursor cells.

This model represents an advance in the state of the art of spatio-temporal bone remodelling models. The model developed by Quexada et al. (2022) accounted for the cyclic and asynchronous cell dynamics of bone remodelling but not for the spatial progression of the process, as can be concluded from the coincidence in time of osteoclasts and osteoblasts within a certain spatial domain. This limitation, along with the short quiescence time between remodelling cycles, is common to the model of Komarova et al. (2003), on which it is based. The model by Ryser et al. (2009) was also based on the same equations and led again to the temporal coincidence of osteoclasts and osteoblasts. Though the latter authors did not explicitly model precursor cells, these were included in the variable that measured the concentration of mature cells, so introducing the concept of cell availability. So, if the concentration of a certain type of cell was below the steady-state value (which acted as a threshold), they were considered precursor cells and the number of cells exceeding that threshold were considered active cells.

Kameo et al. (2020) proposed a diffusion model for signalling molecules that determine the probability of the different cell genesis. Thus, cells appeared randomly on the bone surface without a clear spatio-temporal pattern, in contrast to what occurs in BMUs. A similar behaviour was predicted by the agent-based model developed by Tourolle et al. (2021). Buenzli et al. (2011) developed a 1D bone remodelling model of cortical bone that included the diffusion of cells and signalling molecules across the resulting resorption cavity and refilling cone. However, the movement of the cells was controlled by the growth of the capillary that supplied precursor cells and nutrients, which was imposed *a priori*. This was equivalent to enforce the movement of osteoclasts, but the movement of osteoblasts and their precursors had to be prevented in order to achieve a meaningful spatial pattern of cells within the BMU. Later, these authors extended their model to a 2D agent-based lattice model (Buenzli et al., 2012a), but only the resorption process was simulated and the capillary growth had to be still imposed in order to obtain elongated BMUs. The current model overcomes most of those limitations and the spatio-temporal pattern of the BMU is now achieved without enforcement, basically through the concept of cell availability and the role of TGF-β in intercellular interactions.

### 4.1 Homeostasis

Our results demonstrated that the spatio-temporal BCPM model is able to reach a homeostatic state in a 3D spatial domain, characterised by a slightly oscillating variation of *f*_*bm*_ which remains constant on average (see Figure 5A) due to an equilibrium between the resorption and the formation cycles of BMUs. This is opposed to the monotonic and smooth way the previous temporal-only model reaches a homeostatic state after a parameter perturbation. The oscillations are more realistic since bone turnover events occur sporadically and spread out across bone sites, as was demonstrated in our model simulations, but the amplitude of the oscillations diminishes with the size of the domain where the results are averaged.

It is important to note that during the first phase of the simulation shown in Figure 5, the average *f*_*bm*_ is adapting to the external load, which is too low for the initial distribution assumed for *f*_*bm*_. This leads to a global bone loss during a relatively long transient period where fluctuations of *f*_*bm*_ are pronounced. These fluctuations are damped out in the long-term, showing that the model is able to reach an equilibrium state adapted to the applied load. The amplitude of the oscillations and the length of the transient period depend on the applied load compared to the homeostatic load and on the initial distribution of the variables. In order to consider a situation more realistic than the uniform distribution, some variables were randomly distributed in space. In the case of damage and concentration of osteoclast precursors the amplitude of this perturbation was based on the normal values achieved with the previous BCPM model (Martínez-Reina et al., 2021b). The perturbation of damage was quickly damped, having no effect in the mid-term. The perturbation of other variables such as mineral content also damp out very quickly, so they were not included in these simulations. The perturbations of Oc_p_ and specially of *f*_*bm*_ have a stronger effect since they make BMU activations be more uniformly distributed over time throughout the domain under study. The transient period will be left out from the rest of the discussion and we will focus on the equilibrium state.

Starting from an almost uniform distribution of *f*_*bm*_ (in the range [27, 29]% in Figure 6) leads to an almost synchronous activation of BMUs with large fluctuations of *f*_*bm*_, which is not realistic. This initial distribution of *f*_*bm*_ is also unrealistic from another point of view, as it cannot correctly represent the spatial variation of porosity throughout the trabecular microstructure with elements as small as those of 60 *μ*m in size. In conclusion, an appropriate initial distribution of *f*_*bm*_ is crucial to obtain realistic results and should be chosen according to the element size.

The proposed algorithm sorts the IPs based on the precursors concentration, Oy_p_. As explained in Figure 2 a gradient of Oc_p_ could prevent the activation of BMUs in a spurious way. For instance, consider a monotonic gradient of Oc_p_ in *z*-direction of the model (see Figure 3). If the order of calculation of IPs started in the growing direction of Oc_p_, BMUs would only be activated at the end section and therefore, the activation frequency would be highly underestimated, though in a spurious way. This monotonic gradient of Oc_p_ is not realistic and in fact it is difficult to achieve with the random assignment of initial Oc_p_. With this random assignment, the activation frequency is just slightly underestimated, but this situation must be prevented in any case and for that reason sorting the IPs in the algorithm is important. Only in this case, BMU activation is allowed or prevented depending on the concentration of precursors, in the IP and the surroundings, and not influenced by the order of calculation of IPs.

Figure 5B allowed to compare the temporal evolution of *f*_*bm*_ and the stimulus *ψ*_*bm*_. It could be seen how the minima of *f*_*bm*_ coincide with the maxima of *ψ*_*bm*_. As the resorption phase progresses and *f*_*bm*_ decreases in a certain region, the remaining tissue becomes overloaded and the mechanical stimulus (SED) rises, thus promoting the proliferation of osteoblast precursors and eventually the onset of the formation phase. During this phase that trend is reversed as the rise of *f*_*bm*_ increases the stiffness, so reducing the strains and consequently the SED (note that the stress is kept constant). During the quiescence phase (*f*_*bm*_ ∼ constant) the slow fall of *ψ*_*bm*_ follows the gradual increase in stiffness caused by the mineralisation of the newly formed tissue.

The different phases of a BMU are simulated with the current model in a realistic manner. First, the BMU is activated at a certain location with the differentiation of osteoclast precursors into mature osteoclasts, so starting a resorption cycle aimed at repairing the damaged tissue. The highest peaks observed in Figure 7 correspond to the resorption cycles, which are higher and much shorter than the formation cycles, so revealing that resorption is faster than formation but it extends over a shorter period of time. The new model proved also valid to simulate the reversal phase, so separating the resorption and formation events. Thus, once osteoclasts have finished the resorption phase at a site, they move or undergo apoptosis and the reversal phase takes place before the formation phase begins. Finally, the formation cycle ends and the remodelled region remains in a quiescence state for quite a long time (around 1.5–2 years) until a new BMU is activated. The areas under the RBV and FBV curves are equal, so indicating that the resorbed volume in a cycle is equal to the formed volume (homeostasis). Moreover, the thresholds introduced in the BCPM model were sufficient to obtain a set of phase lengths similar to those found in the literature (see Table 2).

As stated in the introduction, the reversal phase can last from 8–9 days (Eriksen et al., 1984; Hazelwood et al., 2001) to several weeks (Delaisse, 2014; Martin, 2021). The discrepancy in these experimental results may be due to a different interpretation of the reversal phase, with the latter studies including in it the mineralisation lag time. Similar discrepancies exist also in the length of the formation period, with values ranging from 64 (Hazelwood et al., 2001) to 174 days (Eriksen et al., 1990). Even in the latter study the range between the 10% and the 90% percentiles spans 74–481 days. Motivated by these discrepancies, we have used here the values provided by Hazelwood et al. (2001) for comparison of the average times obtained for the resorption, formation and reversal periods, as these authors compared many histomorphometric studies.

Some histomorphometric studies calculated the BMU activation frequency as the inverse of the total time of the BMU cycle. i.e., the summation of the resorption, reversal, formation and quiescence periods (Eriksen et al., 1990; Steiniche et al., 1994). Thus, as the results obtained in this work for those periods are similar to those given the literature, so is the activation frequency measured in this manner. The 3D activation frequency was also calculated here, although it is not possible to compare it with any experimental results. Taking into account the relationship between the 2D and the 3D activation frequencies proposed by Hernandez et al. (1999), our result would be in agreement with the values provided by Frost (1964). This author measured the evolution of BMUs activation rate with age, obtaining values between 1 and 2 BMUs/year/mm^2^ for people over 30 years old. The mean value obtained in this work is in that range; however, it is important to note that the relationship between the 2D and 3D frequencies is not absolutely reliable because the parameters were estimated with a high degree of uncertainty, as stated by the authors (Martin, 1984; Hernandez et al., 1999).

The sensitivity analysis whose results were given in Table 3 shows that $Oc_{p}^{up,th}$ is the threshold with the greatest sensitivity. It affects significantly the inversion and quiescence times and to a lesser extent the formation time and the activation frequency. It can be noted that $Ob_{p}^{\mathrm{low,th}}$ has only a slight influence on the average phase lengths, as does $Oc_{p}^{\mathrm{low,th}}$ , except maybe for the reversal time. $Ob_{p}^{up,th}$ has a high influence on the reversal time, while the rest of times and the activation frequency remain roughly unaffected, except if $Ob_{p}^{up,th}$ is increased by 20% or more. It could be checked that the abnormally high values of *T*_*I*_ were caused by the same lack of coordination of the BMU cycles observed in the simulations with no TGF-β. The radius of the sphere defining the neighbourhood, *R*, has a strong influence on the 3D activation frequency: the larger the radius, the smaller the 3D activation frequency. Certainly, as *R* increases, new activations are prevented in a larger volume if one BMU is already active within the neighbourhood. Finally, both the thresholds and *R* seem to have a small influence on the length of the resorption period.

The influence of *R* on the activation frequency is related to the features of the FE mesh, particularly to the element size and the integration scheme (reduced vs. full integration). If these are changed, the suitable *R* may also change. The reason for this is that both variables also affect the IPs contained within the neighbourhood. In fact, the most influential factor on the activation frequency is the distance from the centre of the neighbourhood to the first IP outside it, where the BMU activation is no longer prevented.

TGF-β is a cytokine stored in the bone matrix and released by osteoclasts during bone resorption, which has been reported to play a crucial role in the coupling formation to resorption (Durdan et al., 2022). It is known that this cytokine promotes osteoclast apoptosis and differentiation of uncommitted osteoblast into osteoblast precursors, while it downregulates the differentiation of osteoblast precursors into mature osteoblasts (Pivonka et al., 2008). In other words, TGF-β promotes the accumulation of osteoblast precursors, so preparing the upcoming formation cycle. The upregulation of osteoclasts apoptosis also helps bring the ongoing resorption cycle to an end. Therefore, TGF-β seems to be of paramount importance in the synchronization of the BMU cycle. We have shown with our model that in the presence of TGF-β BMU cycles exhibit a normal pattern, with resorption followed by formation after a reversal phase and a long quiescence period before the next resorption cycle (see Figure 7). Moreover, the average time for each phase is in agreement with the literature. However, in the absence of TGF-β (see Figure 8), the BMU cycles become completely uncoupled. Resorption and formation would take place simultaneously at a given bone site, which would not have any biological sense. The reversal phase could be extremely long and even formation or resorption cycles could appear isolated. Moreover, these rare events are not one-off. On the contrary, they occur very frequently, the normal cycles being the less usual ones, as can be seen in Table 4.

The comparison of Figures 7, 8 might lead one to think that the equilibrium of *f*_*bm*_ is not achieved in the absence of TGF-β, as the areas under the FBV and RBV curves are not equal. However, it should be noted that the evolution of Figure 7 is not representative of what occurs in average, but only of this particular element and time window. If *f*_*bm*_ is averaged in the domain under study over time, an approximately constant $f_{bm}^{mv}$ is also obtained in the equilibrium, though with larger and more chaotic oscillations than those observed in Figure 5.

As stated before, a lack of coordination of the BMU cycles was also observed in some cases of the sensitivity analysis where *T*_*I*_ was abnormally high (see Table 3). However, the percentage of normal cycles was much higher in these cases and more importantly, that lack of coordination can be avoided with a proper choice of the thresholds, something that is not possible when TGF-β is absent.

Another phenomenon that is better described in intermittent models is the absorption of bisphosphonates. These drugs are also stored in the bone matrix and released through bone resportion, like TGF-β, though their effect is different and seems to affect only osteoclast by impairing its resorptive capacity (Halasy-Nagy et al., 2001; Takagi et al., 2021) and enhancing its apoptosis rate (Sato et al., 1991; Halasy-Nagy et al., 2001; Yu et al., 2018; Takagi et al., 2021). In a recent work we have developed a pharmacokinetics-pharmacodynamics (PK-PD) model to simulate the absorption of alendronate and its effect on bone turnover. This model implements a continuous BCPM and therefore, it does not allow to simulate well-known features of alendronate such as its higher deposition rate at sites where bone turnover is more intense (Porras et al., 1999). This is feasible in spatio-temporal models such the one proposed here as remodelling sites are easily distinguished from quiescent sites. This would lead to a heterogeneous distribution of the drug that could also allow to consider other important effects, such as the saturation of the drug absorption in certain sites, which is not feasible in temporal-only BCPM. Analogously, the heterogeneous distribution of mineral within bone matrix also emerges from the specific localisation of the remodelling events and therefore, it can be addressed by spatio-temporal bone remodelling models, but hardly by temporal-only models.

We have simulated the activation, progression and deactivation of BMUs in a 3D FE model of a piece of trabecular bone with *f*_*bm*_ ∼ 30% under different load cases. This bone volume fraction was selected based on the experimental data taken to compare the quiescence time that was measured in cancellous bone (Steiniche et al., 1994). Trabecular thickness is approximately 90 *μ*m (Vesterby et al., 1991) and the element size of the FE mesh is 60 *μ*m. Therefore, modelling the porous bone as a continuum allows the BMUs to move in any direction, whereas in reality they could only progress onto the existing bone tissue, being forced to turn to follow the trabecula, something that does not occur in our model, which does not account for the microstructure of the trabecular bone. This requires a more complex model with geometrical constraints to the movement of the BMUs and is left for future works.

### 4.2 Targeted bone remodelling

The BCPM presented in this work is able to model the activation of BMUs in highly damaged areas and to steer them to follow a crack (or a region with high damage) in order to repair that damage, as the theory of targeted bone remodelling holds. It is important to note that not several but only one BMU was activated to follow the crack path. The model was designed to avoid the multiple activation of BMUs in a small region, which would not be meaningful from a biological or metabolic point of view. This means that osteoclasts continue to be recruited to the existing BMU if the mechanobiological stimulus (the presence of damage in this case) is still high enough.

As presented in the results of the crack simulation, the BMU repairs only 20%–25% of the existing damage. This result is not very realistic from a biomechanical point of view and this constitutes a limitation of the study. Indeed, osteoclasts dissolve bone mineral by secreting acids and degrade the organic matrix with specialized proteinases (Blair, 1998). If the resorption front spanned the entire damaged region, the resulting resorption cavity would be a void within the mesh the size of several elements, similar to the voids that may appear implementing the element killing technique. This can lead to numerical issues in our FE simulations, arising from the zero stiffness implied by the total removal of bone tissue and was disregarded here. Our model cannot contemplate resorption cavities as such and treats porosity in the Continuum Mechanics sense, as a variable that represents the volume occupied by pores within the elements, without representing the pores explicitly. Similar to that, damage is also considered in the Continuum Mechanics sense, as a measure of microcracks density. Such model is unable to repair damage completely and for this reason, it would be more appropriate to treat this case as the remodelling of a region of high diffuse damage rather than a crack.

## 5 Conclusion

In this work, we have adapted a previously developed temporal-only and local BCPM with modifications affecting the bone cell availability. This new model was implemented in a 3D spatial domain and simulated *via* FE modelling and with it we were able to simulate the intermittent activation of BMUs and its progression along the mesh with the phases of the BMU process well differentiated and several remodelling events spread throughout the piece of bone.

A new homeostasis state is reached after a small perturbation of the equilibrium that includes an underload state, though small oscillations of *f*_*bm*_ are observed due to the intermittent BMU cycles. The shape of the resorption and formation cycles obtained with the model is quite accurate.

The lag time between the resorption and formation phases (reversal phase) arises naturally from the model because of two reasons: 1) *via* TGF-β, which proved to have a key role in the coupling of resorption to formation and 2) the thresholds implemented to activate and deactivate the differentiation from precursors to mature cells, which is an intermittent process in this model.

The average lengths of the BMU phases (resorption, reversal, formation and quiescence) obtained in this work are similar to those found in the literature. Using the neighbourhood concept and limiting the activation of a BMU inside that neighbourhood while an active BMU already exists in it allows to adapt the local model to a non local one, which is able to simulate the progression of BMUs and to yield an average activation frequency which is in accordance with the literature.

## Data Availability

The original contributions presented in the study are included in the article/Supplementary Material, further inquiries can be directed to the corresponding author.

## Footnotes

1 It must be noted that the algorithm is implemented at each integration point of the mesh, as usual in FE formulations.

2 This implies that the metabolic cost is only preventing a further activation in the neighbourhood of the front.

3 If *P* ^TGF−β^ = 0 is also assumed, this stationarity condition leads to $\left[TGF-\beta \right]=\frac{\alpha_{\text{TGF}-\beta }\cdot k_{\mathrm{res}}\cdot Oc_{a}}{{\tilde{D}}_{\text{TGF}-\beta }}$ .

## Abbreviations

BCPM, bone cell population model; BMP-2, bone morphogenetic protein; BMU, basic multicellular unit; BR, bone remodelling; FBV, formation bone volume; FE, finite element; FS, formation state; IGF-1, insulin-like growth factor; IP, integration point; MES, minimally effective strain; NBH, neighbourhood; OPG, osteoprotegerin; PDE, partial differential equation; PDGF, platelet derived growth factor; PK-PD, pharmacokinetics- pharmacodynamics; RANK, receptor activator of nuclear factor *κ*β; RANKL, receptor activator of nuclear factor *κ*β ligand; RBV, resorbed bone volume; RS, resorption state; Runx2, runt-related transcription factor 2; RVE, representative volume element; SED, strain energy density; TGF-β, transforming growth factor *β*; VEGF, vascular endothelial growth factor.
