# Supplementary material for: Spatio-temporal simulations of bone remodelling using a bone cell population model based on cell availability
Source: Front Bioeng Biotechnol. 2023 Mar 7;11:1060158. doi: 10.3389/fbioe.2023.1060158 (PMC10027742; doi:10.3389/fbioe.2023.1060158)
Supplement: Supplementary file 1 [file DataSheet1.PDF]

Supplementary Material to: Spatio-temporal simulations of bone remodelling using a bone cell population model based on cell availability

José Luis Calvo-Gallego[a,*], Pablo Manchado Morales[a], Peter Pivonka[b], Javier Martínez-Reina[a]

[a]*Departamento de Ingeniería Mecánica y Fabricación, Universidad de Sevilla, Seville 41092, Spain*

[b]*School of Mechanical, Medical and Process Engineering, Queensland University of Technology, QLD 4000, Australia.*

---

1. Introduction

In this document we provide those details of the bone cell population model (BCPM) not provided in the main document. More precisely, we explain here those features of the previous model [1] which remained unchanged; while those that have been modified or are more relevant to the study are presented in the main document.

This document is structured as follows. In Section 2 we present the equations of the concentration of the biochemical regulatory factors and their activation and repression functions. The mechanical feedback regulation is explained in Section 3. Section 4 describes how microstructural damage is estimated. The definitions of bone apparent density and tissue density and its relation with stiffness are given in Section 5. The algorithm of bone mineralisation is explained in Section 6 and in Section 7 the degradation of fatigue properties with mineral content is outlined. In Section 8, the model constants are given in Table 1.

*Corresponding author: joselucalvo@us.es

Email addresses: `pabmanmor@gmail.com` (Pablo Manchado Morales), `peter.pivonka@qut.edu.au` (Peter Pivonka), `jmreina@us.es` (Javier Martínez-Reina)

2. Biochemical regulatory factors

2.1. TGF–β

TGF–β is stored in the bone matrix and released during resorption by osteoclasts. Its concentration is calculated following Pivonka et al. [2]:

$$TGF-\beta = \frac{\alpha_{TGF-\beta}\, k_{res}\, Oc_a}{\tilde{D}_{TGF-\beta}} \tag{1}$$

where $\alpha_{TGF-\beta}$ is the concentration of TGF–β in bone matrix and $\tilde{D}_{TGF-\beta}$ is the TGF–β degradation rate. The concentration of TGF–β is used to define the activator/repressor functions in Eqs. 1, 2 and 4 in the main document:

$$\Pi_{act}^{TGF-\beta} = \frac{TGF-\beta}{K_{act}^{TGF-\beta} + TGF-\beta} \tag{2}$$

$$\Pi_{rep}^{TGF-\beta} = \frac{K_{rep}^{TGF-\beta}}{K_{rep}^{TGF-\beta} + TGF-\beta} \tag{3}$$

$K_{act}^{TGF-\beta}$ and $K_{rep}^{TGF-\beta}$ being the activation and repression constants, respectively.

2.2. PTH

The activator/repressor functions that govern PTH regulation on the RANKL-RANK-OPG signalling pathway (Eqs.(7) and (9)) are given by:

$$\Pi_{act}^{PTH} = \frac{PTH}{K_{act}^{PTH} + PTH} \tag{4}$$

$$\Pi_{rep}^{PTH} = \frac{K_{rep}^{PTH}}{K_{rep}^{PTH} + PTH} \tag{5}$$

where the concentration $PTH = 2.91$ pM has been assumed constant in this case [2] and K_{act}^{PTH} and K_{rep}^{PTH} are the corresponding activation and repression constants.

2.3. RANK-RANKL-OPG signaling pathway: competitive binding

The RANK-RANKL-OPG signalling pathway controls the differentiation of uncommitted osteoclast progenitors and osteoclasts maturation through Π_{act}^{RANKL} (see Eqs.3 and 4 in the main document). Thus, an imbalance in that pathway, such as that occurring after menopause, may result in the development of osteoporosis. RANK, expressed by osteoclasts precursors, competes with OPG for binding to RANKL. Following Pivonka et al. [2–4], the concentration of RANK, OPG and RANKL are given by the following equations:

$$RANK = R_{RANK} \cdot Oc_p \tag{6}$$

where R_{RANK} is the RANK carrying capacity of osteoclast precursors. Concentration of OPG is downregulated by PTH:

$$OPG = \frac{\beta_{OPG}\, Ob_a\, \Pi_{rep}^{PTH}}{\tilde{D}_{OPG} + \frac{\beta_{OPG}\, Ob_a\, \Pi_{rep}^{PTH}}{OPG_{max}}} \tag{7}$$

where β_{OPG} and $\tilde{D}_{OPG}$ are, respectively, the production and degradation rates of OPG, while Π_{act}^{PTH} and Π_{rep}^{PTH} are the activator/repressor functions that govern PTH regulation on the RANKL-RANK-OPG signalling pathway (defined in subsection 2.2). The concentration of RANKL is given by:

$$RANKL = RANKL_{eff} \frac{\beta_{RANKL} + P_{RANKL}}{\beta_{RANKL} + \tilde{D}_{RANKL}\, RANKL_{eff}} \cdot \\ \cdot \left[1 + \frac{OPG}{K_{d,[RANKL-OPG]}} + \frac{RANK}{K_{d,[RANKL-RANK]}}\right]^{-1} \tag{8}$$

where $K_{d,[RANKL-OPG]}$ and $K_{d,[RANKL-RANK]}$ are the equilibrium dissociation constants for the binding of OPG and RANK to RANKL. $\tilde{D}_{RANKL}$ is the RANKL degradation rate. P_{RANKL} provides the RANKL production rate that can be induced for example by postmenopausal osteoporosis (PMO) or mechanical underloading. β_{RANKL} is the production rate of endogenous RANKL on the surface of osteoblasts precursors and osteocytes. We have assumed that RANKL is expressed by those cells, following experimental

evidence [5, 6] and a previous model [7]. So, $RANKL_{eff}$ is the total effective carrying capacity of those cells that controls the maximum expression of RANKL:

$$RANKL_{eff} = R_{RANKL} \cdot Ob_p \cdot \Pi_{act}^{PTH} + R_{RANKL} \cdot Ot \cdot \Pi_{act}^{dam} \tag{9}$$

where R_{RANKL} is the RANKL carrying capacity of the individual cells of both types, that we have assumed equal. We have also assumed that the expression of RANKL on the surface of osteoblast precursors is upregulated by PTH, following previous models [2, 3], and by osteocytes due to damage, through a sigmoidal function following Martínez-Reina et al. [1]:

$$\Pi_{act}^{dam} = \frac{d^{\xi}}{d^{\xi} + \delta_{50}^{\xi}} \tag{10}$$

where d denotes the damage variable, described more in detail later in Section 4. The shape factor, $\xi = 3$, and the value of damage leading to a 50% of the maximum response, $\delta_{50} = 0.1$, were chosen in [1] such that the two terms of Eq.(9) are typically of the same order of magnitude. Verborgt et al. [8] showed that osteocyte apoptosis occurs after fatigue-induced bone matrix damage. Moreover, they found that osteocyte apoptosis was highly localised to sites of microdamage that are subsequently remodelled. Osteocytes in the vicinity of a microcrack would express both Bax (a proapoptotic gene product) and Bcl-2 (an antiapoptotic gene product), with the peak of Bax expression observed immediately at the microcrack locus and the peak of Bcl-2 expression at some distance (1–2 mm) from microcracks [9]. Seemingly, distant osteocytes would protect themselves from matrix injury–induced cell death, thereby exercising an additional level of control in the regulation of osteocyte apoptosis and bone remodelling. This expression of apoptotic signals would be related to the expression of *come and eat me* signals [10] to attract macrophages to the site of apoptotic osteocytes. Kurata et al. [11] showed later that focal wounding of osteocyte-like cells (MLO-Y4) in vitro triggered release of RANKL and macrophage-colony stimulating factor (M-CSF), although whether these key signalling molecules come from dying cells or the non-apoptotic surviving cells was not examined. Here we have assumed by using Eq.(9) that apoptotic osteocytes near microcracks would express RANKL thus leading to a subsequent resorption of the surrounding bone matrix.

The RANKL production rate, P_{RANKL}, in Eq.(8) is given by two terms that define the contribution of mechanical underloading, P^{mech}_{RANKL}, and a disease-related (PMO) increase in RANKL production over time, P^{PMO}_{RANKL}:

$$P_{RANKL} = P^{mech}_{RANKL} + P^{PMO}_{RANKL} \tag{11}$$

The first term is explained later in Section 3, while the term P^{PMO}_{RANKL} equals 0 in this work as PMO is not simulated. Finally, the activator function of RANKL in Eqs.3 and 4 in the main document can be expressed as:

$$\Pi^{RANKL}_{act} = \frac{RANKL \cdot RANK}{K_{d,[RANKL-RANK]} + RANKL \cdot RANK}; \tag{12}$$

3. Mechanoregulation

The model includes the mechanical feedback regulation of bone through the Mechanostat Theory proposed by Frost [12] (see Fig.1). This theory postulates the existence of 4 zones or "windows" in Frost's terminology: 1) disuse window, where net bone loss is observed for a low level of "Minimally Effective Strains" (MES) or other stimuli ; 2) adapted window, where no net effect of BMUs on bone mass is seen for intermediate values of MES; 3) mild overload window, where net bone formation occurs for high MES and 4) pathologic overload window, leading to fracture, for very high values of MES. This last window is not directly considered in the mechanical regulation through the definition of $\Pi^{\psi_{bm}}_{act}$ (see Fig.2), but indirectly through the accumulation of microstructural damage.

Mechanical disuse is assumed to enhance the production of RANKL on osteoblasts precursors, through the term P^{mech}_{RANKL}, which is modulated by the strain energy density (SED) of bone matrix, designated as ψ_{bm}:

$$P^{mech}_{RANKL} = \begin{cases} P^{mech,max}_{RANKL} \left(1 - \frac{\psi_{bm}}{\psi_r}\right) & \text{for} \quad \psi_{bm} < \psi_r \\ 0 & \text{for} \quad \psi_{bm} \geq \psi_r \end{cases} \tag{13}$$

where ψ_r is the SED below which underuse increases RANKL production and $P^{mech,max}_{RANKL}$ is the maximum RANKL production rate due to underuse. RANKL production is upregulated by PTH and downregulated by nitric oxide (NO), which is produced by osteocytes and, in turn, upregulated by mechanical stimulus. However, this process is only indirectly considered

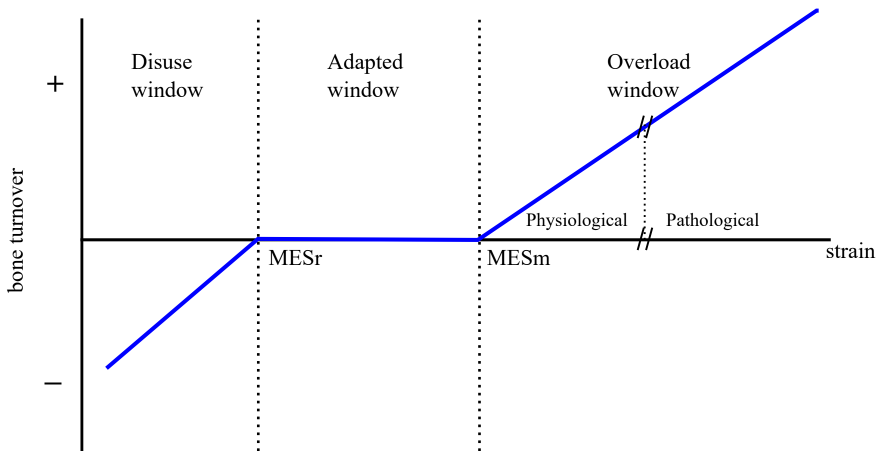

Figure 1: The Mechanostat Theory (adapted from Frost [12]).

through Eq.(13), which assumes a maximum RANKL production rate for total disuse.

Overload is assumed to promote bone formation by proliferation of osteoblast precursors through the activator function $\Pi_{act}^{\psi_{bm}}$, which is given by the piecewise linear function of SED defined in Fig.2. The less steep part of the function would correspond to the disuse and adapted windows of the Mechanostat Theory, where bone formation is not particularly promoted. In the case of the disuse window, this would be added to the increased RANKL production (P_{RANKL}^{mech}). Obviously, the steepest part of function $\Pi_{act}^{\psi_{bm}}$ would correspond to the overload window.

The SED, termed here ψ_{bm}, was used as a measure of the mechanical stimulus sensed by bone cells to drive bone adaptation, as traditionally done in the literature [13, 14]. ψ_{bm} was used here as an alternative to the strains MES, used in the Mechanostat Theory [12]. In a uniaxial stress state both variables are related through:

$$\psi_{bm} = \frac{1}{2} E \cdot MES^2 \tag{14}$$

E being the Young's modulus. The parameter ψ_r in Eq.(13) as well as $\psi_{bm\,1}$ and $\psi_{bm\,2}$, used in function $\Pi_{act}^{\psi_{bm}}$ (see Fig. 2), were defined using Eq.(14), respectively with $MES_r = 1000\,\mu\varepsilon$, $MES_1 = 800\,\mu\varepsilon$ and $MES_2 = 1600\,\mu\varepsilon$. These values and the values of $\Pi_{act}^{\psi_{bm}}$ corresponding to $\psi_{bm\,1}$ and $\psi_{bm\,2}$ were

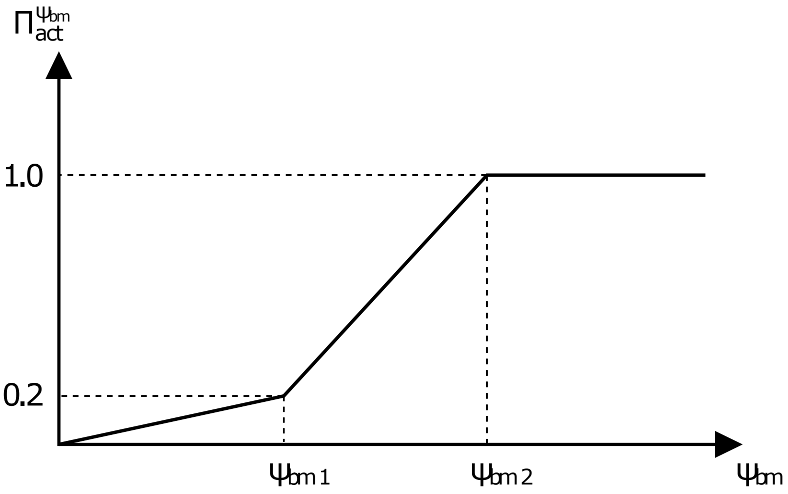

Figure 2: Function of proliferation of osteoblast precursors that establishes the relation between the anabolic factor $\Pi_{act}^{\psi_{bm}}$ and the SED.

adjusted in [1] to reproduce the Mechanostat Theory along with the Principle of Cellular Accomodation [15].

4. Damage of bone matrix

In this section we address how to estimate the microstructural damage, that we assumed to drive bone remodelling through Eq. (9) and to affect mechanical properties as will be discussed shortly. It has long been hypothesised that one of the major functions of bone remodelling is to remove microcracks from the bone matrix so to avoid accumulation of the latter, which could result in macroscopic failure. One way to describe the accumulation of microcracks in a representative volume element (RVE) is via use of Continuum Damage Mechanics [16]. The latter theory introduces a damage variable, d, which is linked to the density of microcracks in a volume of material and to the loss of stiffness through Eq. (15). This variable is such that $d \in [0, 1]$, with $d = 0$ corresponding to an undamaged state and $d = 1$ to a local fracture or failure situation:

$$\mathbf{C} = (1 - d)\, \mathbf{C}_0 \tag{15}$$

where $\mathbf{C}$ and $\mathbf{C}_0$ are the stiffness tensors of damaged and undamaged bone, respectively [16].[1]

Microstructural damage accumulates in the bone matrix due to fatigue loading and is repaired by bone remodelling, as osteoclasts resorb the damaged tissue while the osteoid deposited by osteoblasts is initially intact. The evolution law for damage can be expressed as:

$$\dot{d} = \dot{d}_A - \dot{d}_R \tag{16}$$

where $\dot{d}_A$ is the rate of damage accumulation by fatigue loading and $\dot{d}_R$ is the rate of damage removal by bone remodelling. The latter is assessed by assuming that damage is uniformly distributed throughout the RVE. Thus, the amount of damage repaired by remodelling is proportional to the damage present in that volume and to the volume of tissue being resorbed (see Eq.8 in the main document), through the fraction that this volume represents within the bone matrix volume:

$$\dot{d}_R = d \; \frac{k_{res} \cdot Oc_a}{f_{bm}} \tag{17}$$

Damage accumulation is evaluated following the procedure described in [19, 20], which, in turn, is based on the works by García-Aznar et al. [21] and Pattin et al. [18]. Experimental fatigue tests provide the evolution of d with the strain or the stress level and the number of cycles [18], as well as fatigue life, N_f, which is typically given by expressions such as:

$$N_f = \frac{K_i}{\varepsilon^{\delta_i}} \qquad i = c(\text{compresion}),\ t(\text{tension}) \tag{18}$$

where K_i and δ_i stand for constants that are different in tension and compression and ε is the uniaxial strain expressed in $\mu\varepsilon = \mu m/m$. García-Aznar et al. [21] correlated Eq.(18) with the experimental results obtained by Pattin et al. [18] to get: $K_c = 9.333 \cdot 10^{40}$ and $\delta_c = 10.3$ in compression, and $K_t = 1.445 \cdot 10^{53}$ and $\delta_t = 14.1$ in tension. The loss of stiffness E/E_0 was also experimentally measured by those authors as a function of the applied constant strain and the number of cycles. Again, García-Aznar et al. [21],

[1] In the isotropic damage theory, Eq. (15) can be similarly written in terms of the respective Young's moduli, E and E_0, as $E = (1 - d)\, E_0$ [17, 18]. In the following, we will use the latter formulation.

fitted the experimental curves obtained by Pattin and co-workers with the following expressions:

$$d_c = -\frac{1}{\gamma_c}\left[\ln(1 - C_c\,\varepsilon^{\delta_c} N)\right] \tag{19a}$$

$$d_t = 1 - \left[\frac{1}{C_{t2}}\ln(e^{C_{t2}} - C_{t1}\,\varepsilon^{\delta_t} N)\right]^{\frac{1}{\gamma_t}} \tag{19b}$$

where N is the number of cycles and $C_{t2} = -20$ was fitted from the experimental curves along with:

$$\begin{aligned} &\gamma_c = -5.238(\varepsilon - 6100)10^{-3} + 7; \quad C_c = \frac{1 - e^{-\gamma_c}}{K_c} \quad \text{in compression} \\ &\gamma_t = -0.018(\varepsilon - 4100) + 12; \qquad C_{t1} = \frac{e^{C_{t2}} - 1}{K_t} \quad \text{in tension} \end{aligned} \tag{20}$$

In the damage model proposed by Martínez-Reina et al. [20], cracks were assumed to grow normal to the maximum strain direction and only under tensile strains. This allows to apply the model to a general strain state, by replacing ε with the maximum principal strain, ε_{max}. The tests performed by Pattin et al. [18] and fitted with equations (19) were conducted under constant strain. However, they can be applied to a general loading history using the procedure described in [20] and explained next.

Let us assume that, at a given moment, damage is equal to d and a maximum principal strain ε_{max} is applied N cycles in the next step. Let us calculate the increment of damage accumulated by fatigue, Δd_A, after those cycles are applied. Likely, the current damage was not produced by a constant strain ε_{max}, but we can assume that it was so without loss of generality. Then, we can use Eq.(19b) to work out the number of cycles $\tilde{N}$ that would have been needed to reach the current damage d with the current strain ε_{max}.

$$d = 1 - \left[\frac{1}{C_{t2}}\ln(e^{C_{t2}} - C_{t1}\,\varepsilon_{max}^{\delta_t}\tilde{N})\right]^{\frac{1}{\gamma_t}} \qquad \Longrightarrow \qquad \tilde{N} \tag{21}$$

The increment of damage Δd_A would have been reached with the additional N cycles applied at the present step and can be assessed from:

$$d + \Delta d_A = 1 - \left[\frac{1}{C_{t2}} \ln \left(e^{C_{t2}} - C_{t1}\, \varepsilon_{max}^{\delta_t} (\tilde{N} + N) \right) \right]^{\frac{1}{\gamma_t}} \tag{22}$$

This procedure allows working out the increment of damage, Δd_A, but requires that Eq.(16) be rewritten in incremental form and integrated using an explicit integration scheme, as done in [22, 23].

5. Bone apparent density and stiffness

Bone apparent density changes as a consequence of the variation of porosity, accounted by Eq.8 in the main document, and mineralisation. The latter process controls tissue density, ρ_t, given by:

$$\rho_t = \frac{m}{V_{bm}} = \rho_m\, v_m + \rho_o\, v_o + \rho_w\, v_w \tag{23}$$

where m and V_{bm} are, respectively, the mass and volume occupied by bone matrix, while v_m, v_o and v_w stand for the specific volumes of the three phases that compose bone matrix (namely, mineral, organic and water) and ρ_i stand for the corresponding densities. While v_o can be assumed constant, v_m and v_w vary throughout the mineralisation process (see section 6 for more details). Bone apparent density is then given by porosity (or alternatively bone volume fraction) and bone tissue density:

$$\rho = \frac{m}{V_{bm}} \frac{V_{bm}}{V_{RVE}} = \rho_t\, f_{bm} \tag{24}$$

Finally, the bone stiffness is needed to assess SED. In this study, we have assumed that bone tissue is an isotropic material with a Poisson's ratio $\nu = 0.3$ and a Young's modulus given in MPa by the following correlations:

$$E(\rho, d) = \begin{cases} 2014\, \rho^{2.5}\, (1 - d) & \text{if } \rho < 1.2\ g/cm^3 \\ 1763\, \rho^{3.2}\, (1 - d) & \text{if } \rho \geq 1.2\ g/cm^3 \end{cases} \tag{25}$$

These expressions are based on the correlations experimentally obtained by Jacobs [24], which are multiplied by the factor $(1 - d)$, to consider microstructural damage as usually done in Continuum Damage Mechanics [16] (recall Eq. (15)).

6. Algorithm of bone mineralisation

Bone tissue is made up of bone matrix and pores. Thus, the representative volume element, V_{RVE}, can be divided into the bone matrix volume, V_{bm}, and the volume of vascular pores, V_{vas}. In turn, bone matrix volume is divided into inorganic (mineral), organic (mainly collagen) and water phases, respectively designated as V_m, V_o and V_w:

$$V_{RVE} = V_{bm} + V_{vas} = V_m + V_o + V_w + V_{vas} \tag{26}$$

The volume fractions of extravascular bone matrix and vascular pores are respectively given by $f_{bm} = V_{bm}/V_{RVE}$ and $f_{vas} = 1 - f_{bm} = V_{vas}/V_{RVE}$. The mineral content is usually measured by the so-called ash fraction, the ratio between mass of mineral m_m (or ash mass) and dry mass (the sum of inorganic and organic mass):

$$\alpha = \frac{m_m}{m_m + m_o} = \frac{\rho_m V_m}{\rho_m V_m + \rho_o V_o} \tag{27}$$

where density of hydroxyapatite is taken for $\rho_m = 3.2\, g/cm^3$ [25]. Organic phase is mainly composed of type I collagen, but other non-collagenous proteins are also present. Thus, $\rho_o = 1.2\, g/cm^3$ was adjusted to provide a tissue density $\rho_t = 2.1\, g/cm^3$ for the completely mineralised tissue $\alpha = 0.73$ [21].

Specific volumes are defined by: $v_o = V_o/V_{bm}$, $v_m = V_m/V_{bm}$ and $v_w = V_w/V_{bm}$, which implies that $v_o + v_w + v_m = 1$. Then, Eq.(27) can be given in terms of the specific volumes:

$$\alpha = \frac{\rho_m v_m}{\rho_m v_m + \rho_o v_o} \tag{28}$$

Bone tissue density is given by:

$$\rho_t = \frac{m}{V_{bm}} = \rho_m v_m + \rho_o v_o + \rho_w v_w \tag{29}$$

Mineral accumulates by displacing water present in bone matrix [26]. Therefore, the volume ratio of organic phase is assumed constant during the mineralisation process, $v_o = 3/7$ [27]; while the variations of mineral and water volume ratios hold $\Delta v_m = -\Delta v_w$. We have followed Hernandez et al. [26] to assess the increase of v_m with time, by distinguishing the mineralisation lag time; the primary phase, with a linear increase, and the secondary phase, with an exponentially decreasing rate:

$$v_m(t) = \begin{cases} 0 & \text{if} \quad t \leq t_{mlt} \\ v_{m\,prim}\,\dfrac{t - t_{mlt}}{t_{prim}} & \text{if} \quad t_{mlt} < t \leq t_{prim} + t_{mlt} \\ v_{m\,max} - (v_{m\,max} - v_{m\,prim})\, e^{-\kappa\cdot(t - t_{prim} - t_{mlt})} & \text{if} \quad t_{prim} + t_{mlt} < t \end{cases} \tag{30}$$

where t_{mlt} and t_{prim} are, respectively, the length of the mineralisation lag time and the primary phase; $v_{m\,prim}$ is the mineral specific volume at the end of the primary phase, corresponding to $\alpha = 0.45$ [26]; $v_{m\,max}$ is the mineral specific volume corresponding to the maximum calcium content, $300\,mg/g$ [25]; and κ is a parameter measuring the rate of mineral deposition during the secondary phase (see Table 1). Note that, with the assumptions made above, Eqs. (28) and (29) establish a biunivocal relation between α and ρ_t. Also, according to Eq. (24), apparent density is univocally determined by f_{bm} and α.

The amount of mineral contained in a RVE depends on the age of the tissue through Eq. (30), but the RVE can be made up of tissue patches formed in the recent history, viz. of different ages. Moreover, the tissue within the RVE can be resorbed, which puts the mineral back into the blood flow. The amounts of tissue of different ages contained in the RVE are estimated using the algorithm depicted in Fig. 3 [20]. $\overline{V}_{form}(t,\tau)$ provides the bone volume formed τ days ago and still present (not yet resorbed) at time t. Knowing the distribution of tissue patches of different ages at day t (left column) and the volume formed ($V_{form}(t) = k_{form}\,Ob_a(t)$) and resorbed that day ($V_{res}(t) = k_{res}\,Oc_a(t)$), the distribution at day $t+1$ (right column) can be estimated:

$$\overline{V}_{form}(t+1, i+1) = \overline{V}_{form}(t,i) - V_{res}(t)\,\frac{\overline{V}_{form}(t,i)}{V_{bm}(t)} \tag{31}$$

Finally, the mineral content of each patch is summed to estimate the average mineral content of the RVE at day $t+1$.

$$v_m(t+1) = \frac{\sum_{i=0}^{t_R} \overline{V}_{form}(t+1,i)\cdot v_m(i)}{V_{bm}(t+1)} \tag{32}$$

where the mineral contents of the patches, $v_m(i)$, are calculated through Eq. (30). t_R represents the residence time, e.g. the typical time the patch tissue remains within the bone before being resorbed. This residence time can be

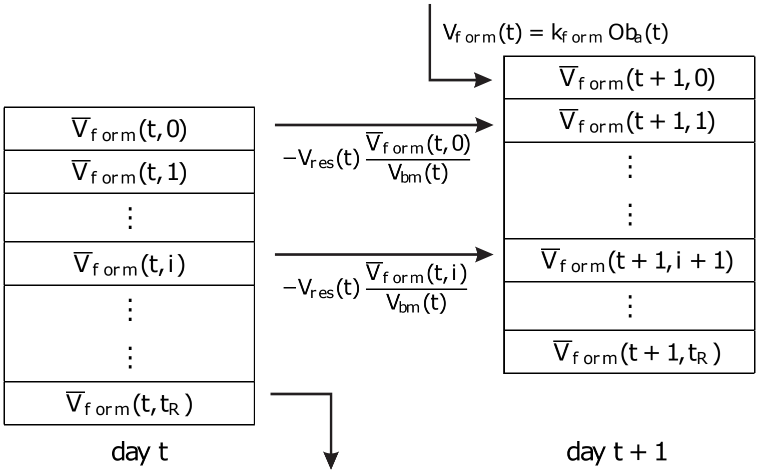

Figure 3: FIFO (first in - first out) queue algorithm used to update the distribution of tissue patches of different ages within the RVE.

very large but the queue can be truncated at a shorter time to reduce the computational cost. See [22] for more details.

It must be noted here that the recursive character of this mineralisation algorithm makes it necessary to integrate the set of differential equations that governs the model using an explicit integration scheme.

7. Degradation of fatigue properties with the mineral content

The mineral phase contributes to increase the stiffness of bone, but also makes it more brittle [25]. As far as we know, no experimental study has provided a correlation between mineral content and bone fatigue properties, though some studies have confirmed that interstitial bone, with the highest mineral content, is where microcracks can be more easily found [28–30]. For this reason, we have followed the damage model previously proposed by Martínez-Reina et al. [20] in which fatigue properties are degraded as the mineral content rises. According to this idea the following assumptions were made in the model (see [20] for more details):

1. The shape of $d - N$ curves, expressed by the equations (19), is maintained regardless of the mineral content.
2. Only the fatigue life is affected by the mineral content, by redefining K_t in equation (18), while keeping constant the exponent δ_t. This modifies

the $d - N$ law, as C_{t1} depends on K_t. Thus, increasing K_t results in a longer fatigue life and a slower damage accumulation rate.

3. A life of 10^7 cycles was assigned to the fatigue limit. This fatigue limit is usually assumed to occur for a given fraction of the ultimate tensile strain, ε_u/β, where the parameter β may depend on the type of material [31]. So, K_t was obtained from equation (18) as:

$$K_t([Ca]) = 10^7 \left(\frac{\varepsilon_u([Ca])}{\beta} \right)^{\delta_t} \tag{33}$$

Here, the most typical value $\beta = 2$ was chosen following Martínez-Reina et al. [20].

4. As Currey showed [25], ε_u depends on the calcium concentration of bone matrix, $[Ca]$. The following regression $\varepsilon_u = \varepsilon_u([Ca])$ was fitted in [20] from the experimental results presented by Currey [25]:

$$log\, \varepsilon_u = 31.452 - 11.341\, log\, [Ca] \tag{34}$$

where ε_u is expressed in $\mu\varepsilon$ and the concentration $[Ca]$ is expressed in mg of calcium per g of bone matrix and is related to the ash fraction, α. More precisely, the relation $[Ca] = 398.8 \cdot \alpha$ was assumed, based on the molecular weigths of hydroxyapatite and type I collagen. Eqs. (20), (33) and (34) allowed to define C_{t1} as a function of α to be used in (19b) and the related equations.

The value fitted by García-Aznar et al. [21], $K_t = 1.445 \cdot 10^{53}$, is assumed here to correspond to a normal value of ash fraction, $\alpha = 0.72$.

8. Values of the constants of the BCPM

The values of the constants of the BCPM are given in table 1. A detailed discussion of these values can be consulted in [1, 22, 32, 33].

Parameter	Value	Units
k_{res}	2	day^{-1}
k_{form}	0.4	day^{-1}
Ob_u	0.01	pM
Oc_p	0.001	pM
D_{Ob_u}	$6.3 \cdot 10^{-4}$	day^{-1}
D_{Ob_p}	$7.89 \cdot 10^{-2}$	day^{-1}
P_{Ob_p}	0.0211	day^{-1}
D_{Oc_u}	$6 \cdot 10^{-3}$	day^{-1}
D_{Oc_p}	0.21	day^{-1}
A_{Ob_a}	0.211	day^{-1}
A_{Oc_a}	0.559	day^{-1}
β_{RANKL}	168.4	pM day^{-1}
$\tilde{D}_{RANKL}$	10.13	day^{-1}
R_{RANKL}	$2.7 \cdot 10^{6}$	-
R_{RANK}	$1 \cdot 10^{4}$	-
β_{OPG}	$1.625 \cdot 10^{8}$	pM day^{-1}
$\tilde{D}_{OPG}$	0.35	day^{-1}
OPG_{max}	$2 \cdot 10^{8}$	pM
$K_{d,[RANKL-OPG]}$	1000	pM
$K_{d,[RANKL-RANK]}$	29,31	pM
η	$4.143 \cdot 10^{-2}$	pM
$P_{RANKL}^{mech,max}$	500	pM day^{-1}
$\tilde{D}_{TGF-\beta}$	2	day^{-1}
$\alpha_{TGF-\beta}$	1	-
$K_{act}^{TGF-\beta}$	$5.633 \cdot 10^{-4}$	pM
$K_{rep}^{TGF-\beta}$	$1.754 \cdot 10^{-4}$	pM
K_{act}^{PTH}	150	pM
K_{rep}^{PTH}	0.223	pM
t_{mlt}	12	days
t_{prim}	10	days
$v_{m\,prim}$	0.121	-
$v_{m\,max}$	0.442	-
κ	0.005	-
t_R	3000	days

Table 1: Values taken for the constants of the BCPM

References

[1] Martínez-Reina J, Calvo-Gallego JL, Pivonka P. Combined effects of exercise and denosumab treatment on local failure in post-menopausal osteoporosis – Insights from bone remodelling simulations accounting for mineralisation and damage. Front Bioeng Biotechnol 2021;9:635056. doi:10.3389/fbioe.2021.635056.

[2] Pivonka P, Zimak J, Smith D, Gardiner B, Dunstan C, Sims N, et al. Model structure and control of bone remodeling: A theoretical study. Bone 2008;43(2):249–63. URL: http://dx.doi.org/10.1016/j.bone.2008.03.025. doi:10.1016/j.bone.2008.03.025.

[3] Pivonka P, Zimak J, Smith D, Gardiner B, Dunstan C, Sims N, et al. Theoretical investigation of the role of the rank–rankl–opg system in bone remodeling. Journal of Theoretical Biology 2010;262(2):306–16. URL: http://www.sciencedirect.com/science/article/pii/S002251930900441X. doi:https://doi.org/10.1016/j.jtbi.2009.09.021.

[4] Pivonka P, Buenzli P, Dunstan C. A systems approach to understanding bone cell interactions in health and disease. In: Gowder SJT, editor. Cell Interaction. IntechOpen; 2012,doi:10.5772/51149.

[5] Nakashima T, Hayashi M, Fukunaga T, Kurata K, Oh-Hora M, Feng J, et al. Evidence for osteocyte regulation of bone homeostasis through RANKL expression. Nat Med 2011;17:1231–4. doi:https://doi.org/10.1038/nm.2452.

[6] Xiong J, O'Brien CA. Osteocyte rankl: new insights into the control of bone remodeling. J Bone Miner Res 2012;27(3):499–505. doi:10.1002/jbmr.1547.

[7] Martin M, Sansalone V, Cooper D, Forwood M, Pivonka P. Mechanobiological osteocyte feedback drives mechanostat regulation of bone in a multiscale computational model. Biomech Model Mechanobiol 2019;18(5):1475–96. doi:10.1007/s10237-019-01158-w.

[8] Verborgt O, Gibson GJ, Schaffler MB. Loss of osteocyte integrity in association with microdamage and bone remodeling after fatigue in vivo. J Bone Miner Res 2000;15(1):60–7. doi:10.1359/jbmr.2000.15.1.60.

[9] Verborgt O, Tatton NA, Majeska RJ, Schaffler MB. Spatial distribution of Bax and Bcl-2 in osteocytes after bone fatigue: complementary roles in bone remodeling regulation? J Bone Miner Res 2002;17(5):907–14. doi:10.1359/jbmr.2002.17.5.907.

[10] Jin Z, El-Deiry WS. Overview of cell death signaling pathways. Cancer Biol Ther 2005;4(2):139–63. doi:10.4161/cbt.4.2.1508.

[11] Kurata K, Heino T, Higaki H, Vaananen H. Bone marrow cell differentiation induced by mechanically damaged osteocytes in 3D gel-embedded culture. J Bone Miner Res 2006;21(4):616–25. doi:10.1359/jbmr.060106.

[12] Frost H. Bone's Mechanostat: A 2003 update. The Anatomical Record Part A 2003;275(A):1081–101. doi:https://doi.org/10.1002/ar.a.10119.

[13] Beaupré GS, Orr TE, Carter DR. An approach for time-dependent bone modeling and remodeling–theoretical development. J Orthop Res 1990;8(5):651–61. doi:10.1002/jor.1100080506.

[14] Huiskes R, Weinans H, Grootenboer HJ, Dalstra M, Fudala B, Sloof TJ. Adaptive bone-remodelling theory applied to prosthetic design analysis. J Biomech 1987;20:1135–50. doi:10.1016/0021-9290(87)90030-3.

[15] Turner CH. Toward a mathematical description of bone biology: The principle of cellular accommodation. Calcif Tissue Int 1999;65(6):466–71. doi:10.1007/s002239900734.

[16] Lemaitre J, Chaboche J. Mechanics of Solid Materials. Cambridge, UK: Cambridge University Press; 1990.

[17] Zioupos P. znd Currey J. Changes in the stiffness, strength, and toughness of human cortical bone with age. Bone 1998;22(1):57–66.

[18] Pattin CA, Caler WE, Carter DR. Cyclic mechanical property degradation during fatigue loading of cortical bone. J Biomech 1996;29(1):69–79.

[19] Martínez-Reina J, García-Aznar J, Domínguez J.and Doblaré M. On the role of bone damage in calcium homeostasis. J Ther Biol 2008;254(3):704–12.

[20] Martínez-Reina J, Garía-Aznar J, Domínguez J, Doblaré M. A bone remodelling model including the directional activity of BMUs. Biomech Model Mechanobiol 2009;8(2):111–27.

[21] García-Aznar JM, Rueberg T, Doblaré M. A bone remodelling model coupling microdamage growth and repair by 3D BMU-activity. Biomech Model Mechanobiol 2005;4(2-3):147–67. doi:10.1007/s10237-005-0067-x.

[22] Martínez-Reina J, Pivonka P. Effects of long-term treatment of denosumab on bone mineral density: insights from an in-silico model of bone mineralization. Bone 2019;125(125):87–95. URL: https://www.ncbi.nlm.nih.gov/pubmed/31055117. doi:10.1016/j.bone.2019.04.022.

[23] Martínez-Reina J, Calvo-Gallego J.L. Pivonka P. Are drug holidays a safe option in treatment of osteoporosis?—insights from an in silico mechanistic pk–pd model of denosumab treatment of postmenopausal osteoporosis. J Mech Behav Biomed Mater 2021;113:104140. doi:10.1016/j.jmbbm.2020.104140.

[24] Jacobs C. Numerical simulation of bone adaptation to mechanical loading. Ph.D. thesis; Stanford University; 1994.

[25] Currey J. Tensile yield in compact bone is determined by strain, post-yield behaviour by mineral content. J Biomech 2004;37(4):549–56.

[26] Hernandez C, Beaupré G, Carter D. A model of mechanobiologic and metabolic influences on bone adaptation. J Rehabil Res Dev 2001;37(2):235–44.

[27] Martin RB. Porosity and specific surface of bone. Crit Rev Biomed Engl 1984;10(3):179–222.

[28] Boyce TM, Fyhrie DP, Glotkowski MC, Radin EL, Schaffler MB. Damage type and strain mode associations in human compact bone bending fatigue. J Orthopaed Res 1998;16(6):322–329. doi:10.1002/jor.1100160308.

[29] O'Brien FJ, Taylor D, Dickson GR, Lee TC. Visualisation of three-dimensional microcracks in compact bone. J Anat 2000;197(3):413–20. doi:10.1046/j.1469-7580.2000.19730413.x.

[30] Qiu S, Rao DS, Fyhrie DP, Palnitkar S, Parfitt AM. The morphological association between microcracks and osteocyte lacunae in human cortical bone. Bone 2005;37(1):10–5. doi:10.1016/j.bone.2005.01.023.

[31] Juvinall R. Engineering Considerations of Stress, Strain and Strength. New York, USA: McGraw-Hill; 1967.

[32] Marathe DD, Marathe A, Mager DE. Integrated model for denosumab and ibandronate pharmacodynamics in postmenopausal women. Biopharmaceutics and Drug Disposition 2011;32(8):471–81. URL: http://dx.doi.org/10.1002/bdd.770. doi:10.1002/bdd.770.

[33] Scheiner S, Pivonka P, Smith D, Dunstan C, Hellmich C. Mathematical modeling of postmenopausal osteoporosis and its treatment by the anti-catabolic drug denosumab. Int J Numer Method Biomed Eng 2014;30(1):1–27. URL: http://dx.doi.org/10.1002/cnm.2584. doi:10.1002/cnm.2584.
